# A nanobody inhibiting porcine reproductive and respiratory syndrome virus replication via blocking self-interaction of viral nucleocapsid protein

**DOI:** 10.1128/jvi.01319-23

**Published:** 2023-12-12

**Authors:** Hong Duan, Xu Chen, Ziwei Zhang, Zhijie Zhang, Zhihan Li, Xinjie Wang, Jiakai Zhao, Yuchen Nan, Baoyuan Liu, Angke Zhang, Yani Sun, Qin Zhao

**Affiliations:** 1 Department of Preventive Veterinary Medicine, College of Veterinary Medicine, Northwest A&F University, Yangling, Shaanxi, China; 2 College of Veterinary Medicine, Henan Agricultural University, Zhengzhou, Henan, China; 3 Agricultural Genomics Institute at Shenzhen, Chinese Academy of Agricultural Sciences, Shenzhen, China; University of Michigan Medical School, Ann Arbor, Michigan, USA

**Keywords:** porcine reproductive and respiratory syndrome virus, nanobody, nucleocapsid protein, antiviral drug, viral replication

## Abstract

Porcine reproductive and respiratory syndrome (PRRS) is a serious global pig industry disease. Understanding the mechanism of viral replication and developing efficient antiviral strategies are necessary for combating with PRRS virus (PRRSV) infection. Recently, nanobody is considered to be a promising antiviral drug, especially for respiratory viruses. The present study evaluated two nanobodies against PRRSV nucleocapsid (N) protein (PRRSV-N-Nb1 and -Nb2) for their anti-PRRSV activity *in vitro* and *in vivo*. The results showed that intracellularly expressed PRRSV-N-Nb1 significantly inhibited PRRSV-2 replication in MARC-145 cells (approximately 100%). Then, the PRRSV-N-Nb1 fused with porcine IgG Fc (Nb1-pFc) as a delivering tag was produced and used to determine its effect on PRRSV-2 replication in porcine alveolar macrophages (PAMs) and pigs. The inhibition rate of Nb1-pFc against PRRSV-2 in PAMs could reach >90%, and it can also inhibit viral replication *in vivo*. Epitope mapping showed that the motif Serine 105 (S105) in PRRSV-2 N protein was the key amino acid binding to PRRSV-N-Nb1, which is also pivotal for the self-interaction of N protein via binding to Arginine 97. Moreover, viral particles were not successfully rescued when the S105 motif was mutated to Alanine (S105A). Attachment, entry, genome replication, release, docking model analysis, and blocking enzyme-linked immunosorbent assay (ELISA) indicated that the binding of PRRSV-N-Nb1 to N protein could block its self-binding, which prevents the viral replication of PRRSV. PRRSV-N-Nb1 may be a promising drug to counter PRRSV-2 infection. We also provided some new insights into the molecular basis of PRRSV N protein self-binding and assembly of viral particles.

**IMPORTANCE:**

Porcine reproductive and respiratory syndrome virus (PRRSV) causes serious economic losses to the swine industry worldwide, and there are no highly effective strategies for prevention. Nanobodies are considered a promising novel approach for treating diseases because of their ease of production and low costing. Here, we showed that PRRSV-N-Nb1 against PRRSV-N protein significantly inhibited PRRSV-2 replication *in vitro* and *in vivo*. Furthermore, we demonstrated that the motif Serine 105 (S105) in PRRSV-N protein was the key amino acid to interact with PRRSV-N-Nb1 and bond to its motif R97, which is important for the self-binding of N protein. The PRRSV-N-Nb1 could block the self-interaction of N protein following viral assembly. These findings not only provide insights into the molecular basis of PRRSV N protein self-binding as a key factor for viral replication for the first time but also highlight a novel target for the development of anti-PRRSV replication drugs.

## INTRODUCTION

Porcine reproductive and respiratory syndrome (PRRS) is one of the most serious global pig industry diseases (1). Currently, there are no highly effective strategies to protect pigs from PRRS virus (PRRSV) infection due to viral variability (2), persistence (3), destruction of lung alveolar macrophages (4), and antibody-dependent enhancement (5). Therefore, improving our understanding of the mechanism underlying viral replication and developing efficient antiviral strategies for combating PRRSV infections in pigs are imperative.

PRRSV, the causative agent of PRRS, is an enveloped single-stranded RNA virus that belongs to the genus *Arterivirus*, family *Arteriviridae*, order *Nidovirales* (6, 7). The complete genome of PRRSV is ~15 kb and contains at least 10 open reading frames (ORFs), including ORF1a, ORF1b, ORF2a, ORF2b, ORF3, ORF4, ORF5, ORF5a, ORF6, and ORF7 (8). The ORF7 gene encodes a nucleocapsid (N) protein with a molecular mass of 15 kDa. The N protein is comprised of 123 and 128 amino acids for North American (genotype 2, PRRSV-2) and European (genotype 1, PRRSV-1) genotypes, respectively, and is relatively well conserved among different PRRSV strains (8). It accounts for ~40% of virion proteins (9) and exhibits multiple functions throughout the viral life cycle (10).

As we know, PRRSV N protein can encapsulate viral RNA genome, maintain tertiary protein structure for viral assembly, and interact with viral non-structural protein 2 (Nsp2) and Nsp9 to participate in viral replication (11–13). In addition, it can also interact with several host factors involved in viral pathogenesis (14, 15). A previous study reported that the N protein could assemble into a sphere of 20–30 nm in diameter in the form of a dimer (16) and that the dimer was an essential structure for binding to the RNA genome and assembly of viral particles (17). Other reports documented that three conserved cysteine residues in the protein formed disulfide linkages and stabilized the spherical structure (16, 17). However, there have yet to be studies on the amino acids or motifs involved in the self-interaction (dimer formation) of the PRRSV N protein.

Nanobodies are derived from camelids’ naturally occurring heavy-chain antibody variable region and are the smallest functional antibody fragments (12–15 kDa) (18). Due to the advantages of easy production, recognition of concave epitopes, and their ability to be easily modified *in vitro*, nanobodies are considered a suitable direction for antibody drug design and a novel tool for studying the functions of proteins (19). PRRSV mainly attacks porcine alveolar macrophages (PAMs) of lung tissue and the Fc portion of IgG as the most effective phagocytic leukocyte receptors. Herein, the nanobody can be fused with porcine IgG Fc (pFcγ) as the delivery tag to enter into PAMs to inhibit viral replication. Additionally, the IgG Fc can activate FcγRs, and then, the phagocytic leukocytes produce a variety of cytokines, chemokines, and lipid mediators for antiviral infection (20, 21). In particular, nanobodies have been investigated as a treatment for respiratory diseases, as they can be administered by inhalation (22, 23). PRRS is a severe respiratory disease in pigs primarily affecting the lung tissue. Therefore, nanobodies may be a potential strategy for targeting PRRSV infection in pigs.

Our previous study showed that two nanobodies against PRRSV N protein (PRRSV-N-Nb1 and -Nb2) were screened from an immunized camel, and they could be blocked binding to PRRSV N protein by anti-PRRSV antibodies in pig sera, indicating that the epitopes recognized by the two nanobodies were present in the PRRSV particles and induced immune responses in pigs (24). Based on these results, whether the two nanobodies could inhibit PRRSV replication and are promising antiviral drugs was assessed in the present study. Thus, the two nanobodies fused with Fc were designed and produced. The results showed that the Nb1-Fc against PRRSV-N protein could significantly inhibit PRRSV replication *in vitro* and *in vivo*. Subsequently, epitope mapping showed that Serine 105 (S105) in the PRRSV-2 N protein was the key amino acid for binding to PRRSV-N-Nb1 and for self-interaction of the N protein via binding to itself R97. It was also found that PRRSV-N-Nb1 could block the self-binding of N protein, thus preventing the assembly of viral particles. These findings provide novel insights into the molecular basis of PRRSV N protein self-interaction and drug targets for designing anti-PRRSV infection.

## MATERIALS AND METHODS

### Cells and viruses

HEK293T and MARC-145 cells (originally sub-cloned from African green monkey kidney MA-104 cells) were maintained in Dulbecco’s modified Eagle’s medium (DMEM; Thermo Fisher Scientific Inc.) supplemented with 10% fetal bovine serum (FBS) (Gibco; Thermo Fisher Scientific Inc.) and 1% penicillin-streptomycin (Thermo Fisher Scientific Inc.). As previously described, PAMs were obtained by postmortem lung lavage of 8-week-old PRRSV-negative pigs (25). PAMs were cultured in RPMI-1640 medium (Gibco, USA) supplemented with 10% FBS and penicillin-streptomycin. All cells were cultured at 37°C with 5% CO_2_.

The full-length cDNA infectious clone of PRRSV SD16 (rSD16) and GFP-PRRSV (GenBank ID: JX087437) was constructed based on a previous study (26). In addition, PRRSV-2 strains NADC30-like (GenBank ID: KX766379), GD-HD (GenBank ID: KP793736), VR2332 (GenBank ID: EF442771), and PRRSV-1 strain GZ11-G1 (GenBank ID: KF0011144) were used for the viral inhibition assay. SD16, NADC30-like, and GD-HD strains are the highly pathogenic PRRSV (HP-PRRSV), and VR-2332 is the classical PRRSV (27). All these PRRSV strains were titrated in MARC-145 cells and stored at −80°C before use.

### Establishment of MARC-145 cell lines stably expressing nanobodies

First, the recombinant MARC-145 cells expressing the nanobodies were constructed to analyze the effects of intracellularly inhibiting PRRSV replication. The genes encoding PRRSV-N-Nb1 and PRRSV-N-Nb2 were separately amplified with primers Nb-F and Nb-R (Table 1) using the pMECS-PRRSV-N-Nb1 and -Nb2 plasmids as the templates (24), respectively. To construct the recombinant plasmids of pTRIP-CMV-Puro^Nb1-HA^ and -Puro^Nb2-HA^, PCR products were digested with the *Xho* I and *BamH* I enzymes (NEB) and inserted into the pTRIP-CMV-Puro vector digested with the same enzymes. After confirmation of insertion by Sanger sequencing, the positive plasmids pTRIP-CMV-Puro^Nb1-HA^ or -Puro^Nb2-HA^ (0.6 µg) and helper plasmids psPAX2 (0.9 µg) and pMD2.0G (0.5 µg) were co-transfected into HEK293T cells to produce pseudotyped lentivirus particles using X-tremeGENE HP DNA Transfection Reagent (Roche Diagnostics GmbH) according to the manufacturer’s instructions. The second pseudotyped lentivirus system was successfully constructed. Subsequently, at 48 h post-transfection (hpt), cell culture supernatants were collected, and then, 5-multiplicity of infection (MOI) lentivirus was transduced into MARC-145 cells supplemented with 1 µg/mL PolyBrene (Sigma-Aldrich; Merck KGaA). At 36 h post-transduction, positive cells were screened by adding into culture mediums 5 µg/mL puromycin (Sigma-Aldrich; Merck KGaA). Surviving cells were analyzed using western blotting to determine the expression of Nb1-HA and Nb2-HA fusion proteins. Negative control cells stably expressing Nb53-HA fusion proteins were also constructed. Nb53 is a negative nanobody that does not bind to the PRRSV-N protein (28). Finally, the cell viabilities of the three recombinant cell lines were evaluated using a Cell Counting Kit-8 (CCK-8) assay (Beyotime Institute of Biotechnology) according to the manufacturer’s instructions as the following descriptions.

**TABLE 1:** Primers used in this study^*a*^

Primer	Sequence (5′−3′)	Usage
Nbx-F	CCG CTCGAGATGCAGGTGCAGCTGCAGGAG	pTRIP-CMV-Puro^Nbx-HA^
Nbx-R	CGCGGATCCTTAAGCGTAATCTGGAACATCGTATGGGTA TGAGGAGACGGT	
Nbx-pFc-F Nbx-pFc-R	GGTGAATTCCAGGTCCAACTGCAGGAGTC GGGTCTAGATCACTTGCCCTGTGTCTTGC	pCMV-Nbx-pFc
N (1-95)-F	CGCGGATCCATGCCAAATAACAACGGCAAGC	pET28a-different truncated N
N (1-95)-R	CCCAAGCTTTGAATCTGACAGGGCACAAGTC	
N (30-123)-F	CGCGGATCCATGATCGCTCAGCAAAACCAGT	
N (30-123)-R	CCCAAGCTTTGCTGAGGGTGATGCTGTGACGC	
N (30-113)-F	CGCGGATCCATGATCGCTCAGCAAAACCAGT	
N (30-113)-R	CCCAAGCTT ACGCACAGTATGTTGCGTCGG	
N (1-102)-F	CGCGGATCCATGCCAAATAACAACGGCAAGC	
N (1-102)-R	CCCAAGCTTCACAGTGTAACTTATCCTCCCT	
N (1-109)-F	CGCGGATCCATGCCAAATAACAACGGCAAGC	
N (1-109)-R	CCCAAGCTTTTGCGTCGGCAAACTAAACTCCA	
GZ11-G1-F	CGCGGATCCATGGCCGGTAAAAATCAGAGC	pET28a-GZ11-G1 N
GZ11-G1-R	CCCAAGCTTATTCGCACCCTGACTGGC	
N^3M^-F	GAGTTTGCCTTGCCGGCCGCCCATACTGT	pET28a-different mutated N
N^3M^-R	ACAGTATGGGCGGCCGGCAAGGCAAACTC	
N^S105A^-F	GGAGTTTGCCTTGCCGAC	
N^S105A^-R	GTCGGCAAGGCAAACTCC	
N^T108A^-F	TTAGTTTGCCGGCCCAACATACT	
N^T108A^-R	AGTATGTTGGGCCGGCAAACTAA	
N^Q109A^-F	TTAGTTTGCCGACGGCCCATACT	
N^Q109A^-R	AGTATGGGCCGTCGGCAAACTAA	
pBAC-AscI-F	TTACTGGAAATGGTGAGGACTG	pBAC-rSD16 mutated infection clones
pBAC-RsrII-R	AGTGGGAGTGGCACCTTCCAGGGTC	
PRRSV ORF7-F	AGATCATCGCCCAACAAAAC	RT-qPCR
PRRSV ORF7-R PRRSV NSP9-F PRRSV NSP9-R	GACACAATTGCCGCTCACTA CCTGCAATTGTCCGCTGGTTTG GACGACAGGCCACCTCTCTTAG	
β-Actin-F	TCCCTGGAGAAGAGCTACGA	
β-Actin-R	AGCACTGTGTTGGCGTACAG	

^*a*^ Restriction sites are underlined.

### Flow cytometry assay

Flow cytometry assay (FCM) was performed to analyze GFP-PRRSV infection in the recombinant MARC-145^Nb1-HA^, MARC-145^Nb2-HA^, and MARC-145^Nb53-HA^ cells. Briefly, after the three recombinant cell lines were cultured in the six-well plates at a density of 2 × 10^5^ cells per well for 24 h, they were separately inoculated with the recombinant GFP-PRRSV at 0.1 MOI. After infection for 2 h, the cells were rinsed three times with phosphate buffer saline (0.01 M PBS, pH 7.2), and DMEM supplemented with 3% FBS was added for culturing. At 36 h post-infection (hpi), the cells were collected, and the numbers of cells with fluorescing green were detected on a FACSCalibur flow cytometer (BD Bioscience, USA). The data were analyzed using the FlowJo software version 7.6 (FlowJo LLC).

### Production of nanobodies fused with the porcine IgG Fc fragment

As we know, PAMs with Fcγ receptors served as the primary host cells for PRRSV infection. The anti-PRRSV-N nanobody, which was stably expressed in the recombinant MARC-145 cells and can significantly inhibit PRRSV replication, was selected and expressed with porcine IgG Fc fragment as a delivering tag into PAMs based on the previous descriptions (29). The genes encoding nanobody-fused pFc (Nbx-pFc) and negative nanobody (Nb53-pFc) were synthesized by GENEWIZ Company (China). To construct the recombinant plasmids of pCMV-Nbx-pFc, the enzymes *BamH* I and *Xho* I were added to the primers to amplify the complete genes encoding the nanobodies with pFc tags (Table 1). Then, the two genes inserted into the pCMV-HA vector were digested with *BamH* I and *Xho* I. After sequencing, the positive plasmids were electro-transformed into HEK293T cells to produce the fusion proteins. The cell supernatants were collected after transfection for 48 h and purified using Protein G resins according to manual instructions (GenScript, China). The expression and purification of the fusion proteins were performed by Jiangsu Huakang Biotechnology (China).

### The affinity of a nanobody with pFc fusion protein binding to PRRSV-N protein

The ELISA was performed to evaluate the binding ability of the Nbx-pFc fusion protein to the PRRSV-N protein. The 96-well ELISA plate was coated with PRRSV-N proteins (400 ng/well) and incubated overnight at 4°C. After blocking and washing, the wells were added with the Nbx-pFc fusion protein (10^−3^ to 10^6^ ng/well) and incubated for 1 h at room temperature (RT). Next, the horseradish peroxidase (HRP)-conjugated goat anti-swine IgG (1:5,000; Jackson ImmunoResearch) was incubated for 1 h at RT as the secondary antibody. The plates were washed again and added with tetramethyl benzidine (100 µL/well). Finally, the 3 M H_2_SO_4_ (50 µL/well) was added to stop the colorimetric reaction, and the OD_450nm_ values were read using an automated ELISA plate reader (BioTek Instruments Inc.).

### Cell viability analysis

To analyze the cytotoxicity of Nbx-pFc fusion proteins, cell viability was evaluated using a CCK-8 assay (Beyotime Institute of Biotechnology) according to the manufacturer’s instructions. Briefly, after the PAMs were cultured in the 96-well plates at a density of 1 × 10^5^ cells per well for 4 h, they were incubated with different concentrations of Nbx-pFc fusion proteins in DMEM supplemented with 3% FBS for 24 h. Then, the CCK-8 reagent (10 µL/well) was added and incubated in 5% CO_2_ at 37°C for 1 h. The OD_450nm_ values were read using an Epoch microplate spectrophotometer (BioTek Instruments, Inc.) to calculate cell viability.

### Analysis of Nb1-pFc entering into the PAMs

The PAMs (1 × 10^6^ cells/mL) were cultured in the 24-well plates for 4 h, and they were incubated with Nb1-pFc, Nb53-pFc, and Nb1-His at concentrations of 5 to 40 µM for 12 h or 40 µM for a set period (1, 2, and 4 h). Finally, the cells were collected or fixed and analyzed using western blotting and IFA.

### Nanobody with pFc fusion protein inhibits PRRSV replication in PAMs

The PAMs (1 × 10^6^ cells/mL) were cultured in the 6-well or 24-well plates for 4 h and treated with Nbx-pFc fusion proteins for 2 h. Then, cells were inoculated with PRRSVs (different strains including GFP-PRRSV, SD16, VR2232, NADC30-like, and GD-HD) at 0.01 MOI. At 36 hpi, cells were infected with the GFP-PRRSV and were imaged using a fluorescence microscope. For SD16, VR2332, NADC30-like, GD-HD-inoculated cells, the cells and supernatants were collected at 24 hpi and 36 hpi to analyze by western blotting, reverse transcription-quantitative PCR (RT-qPCR), and progeny virus titration assay.

### IFA

The PAMs (1 × 10^6^ cells/mL) were plated in a 24-well plate for 24 h and infected with PRRSV of 0.1 MOI. At 24 hpi, they were fixed using 70% ice-cold ethanol for 1 h at 4°C and blocked using 1% BSA for 1 h at RT. Next, cells were incubated with Nbx-pFc and 6D10 mAb against PRRSV N protein for 2 h at RT. Samples were next incubated with secondary antibodies Alexa Fluor 488-labeled goat anti-swine IgG (H&L) (1:500; catalog no. ab150077; Abcam) and Alexa Fluor 594-conjugated goat anti-mouse IgG (H&L) (1:500; catalog no. ab150116; Abcam) for 1 h at 37°C. Finally, the nucleus was stained using 4′,6′-diamidino-2-phenylindole (DAPI) (Thermo Fisher Scientific Inc.) at RT for 10 min. Image acquisition was performed using a fluorescence microscope (Leica Microsystems Inc.).

### Pull-down assay

To further determine whether Nbx-pFc interacted with the PRRSV N protein in the PAMs, the cells were infected with 0.1 MOI PRRSV and harvested at 36 hpi. These PRRSV-infected cells were lysed using NP40 buffer (Beyotime Institute of Biotechnology) for 30 min, and the supernatants were collected by centrifuging at 12,000 × *g* for 10 min at 4°C. Next, the Nbx-pFc and Protein G (30 µL; catalog no. 10004D; Invitrogen; Thermo Fisher Scientific Inc.) were co-incubated for 12 h at 4°C. Subsequently, the Nbx-pFc beads were washed three times with PBS, and cell supernatants were incubated with the beads at 4°C for 12 h. The protein-bound beads were washed three times with PBS again. Finally, the immunoprecipitated proteins were analyzed by western blotting with anti-PRRSV N protein mAb 6D10 as primary antibodies to detect PRRSV-N protein.

### Western blotting

Cells were harvested and lysed using NP40 buffer. The whole cellular proteins (30 µg) were loaded onto a 12% SDS-gel, resolved using SDS-PAGE, and transferred to polyvinylidene difluoride (PVDF) membranes (Millipore Sigma). After blocking for 2 h at RT with 5% skimmed milk in PBS-Tween 20 (0.01% PBST), the PVDF membranes were incubated separately with primary antibodies: mouse anti-HA mAb (1:2,000; ProteinTech Group Inc.), mAb 6D10 (1:1,000), rabbit anti-camel polyclonal antibody (1:1,000) (28), and anti-α-tubulin (1:5,000; catalog no. 66031–1; ProteinTech Group Inc.). HRP-conjugated goat anti-mouse IgG, goat anti-rabbit IgG, or goat anti-swine IgG (1:5,000; Jackson ImmunoResearch) was incubated for 2 h at RT as the secondary antibody, respectively. Finally, the membranes were visualized using an ECL substrate (Solarbio). Chemiluminescence signal acquisition was performed using a ChemiDoc MP imaging system (Bio-Rad Laboratories Inc.).

To verify the epitope recognized by the nanobody, the different truncated and mutated PRRSV-N proteins (2 µg) were designed, expressed, and resolved by SDS-PAGE and transferred to PVDF membranes. After blocking, the PVDF membranes were incubated with nanobody with HRP fusion proteins for 1 h at RT, as previously described (24). Finally, the membranes were visualized using an ECL substrate and observed with the ChemiDoc MP imaging system.

### Reverse transcriptase-quantitative PCR

RT-qPCR was performed to determine the amount of PRRSV RNA from cells (30). Total RNAs were extracted from the infected cells using TRIzol reagent (Invitrogen; Thermo Fisher Scientific Inc.), and 500 ng total RNA was reverse transcribed into cDNA using the PrimeScript RT Reagent Kit (Takara Bio Inc., Dalian, China) following the manufacturer’s instructions. The qPCR was performed using a StepOne Plus Real-Time PCR System (Applied Biosystems; Thermo Fisher Scientific Inc.) and FastStart Universal SYBR Green Master (Roche Diagnostics GmbH) in a 10 µL reaction volume. Reaction conditions were 95°C for 10 min, 95°C for 15 s, and 60°C for 1 min with 40 cycles. Relative expression of the ORF7 or NSP9 gene was calculated using the 2^−ΔΔCT^ method by relative qPCR, with β-actin as the internal control (31). The sequences of the primers used for qPCR are listed in Table 1.

### Virus titration

Viral progeny production was determined by titration as previously described with some modifications (30). Briefly, MARC-145 cells were seeded into 96-well plates (1 × 10^4^ cells per well) and cultured for 24 h. Viral supernatants from the infected cells were 10-fold serially diluted, and 100 µL of the different dilutions was added to each well with eight repeats per condition. A total of 4 days after infection, the cytopathic effect was observed, and the 50% tissue culture infective dose (TCID_50_) was calculated using the Reed-Munch method (30).

### Animal experiments

Fifteen 4-week-old healthy piglets were randomly divided into four groups and separately raised in different isolation rooms. Details of piglet groupings are shown in Table 2. All pigs were acclimated to the environment for 2 days. Then, all the piglets except those in the negative control group were intramuscularly administered with 1 × 10^6.5^ TCID_50_ of HP-PRRSV JXA1 strain on the third day, whereas the control group received the same volume of PBS. At 6 h after the challenge, 2 mg Nb1-pFc or Nb53-pFc (1 mg/mL) was intramuscularly administered to each piglet in the corresponding groups. The same volume of PBS was administered in the control and mock groups using the same injection route. At 24 h and 48 h after treatment, the same dose of nanobodies was given again.

**TABLE 2:** Animal groups and corresponding treatments

Group name	Challenge PRRSV	Injected Nbx-pFc
Control (*n* = 3)	PBS	PBS
Mock/HP-PRRSV (*n* = 4)	JXA1	PBS
Nb53-pFc/HP-PRRSV (*n* = 4)	JXA1	Nb53-pFc
Nb1-pFc/HP-PRRSV (*n* = 4)	JXA1	Nb1-pFc

Observation of clinical symptoms and rectal temperature measurements were performed daily. Necropsy and gross pathological examinations of the lungs were immediately performed once the piglets and the remaining surviving piglets were euthanized and necropsied at 28 dpi.

The lungs were examined for gross pathological changes, and lung tissues were sampled for histopathologic examination to evaluate the protection efficiency of Nb1-pFc. The level of gross lung lesions was also evaluated according to a standard scoring system previously described (32, 33). Each lung lobe, including the anterior, middle, caudal ventral, dorsal aspects, and accessory lobes, was assigned several points (100 points in total). Each piglet’s lung was given a score based on pathological changes in five parts of each lobe to reflect the percentage of the lung showing signs of pneumonia. All lung tissue samples were fixed with 10% formalin and embedded in paraffin blocks. After the blocks were sectioned, hematoxylin and eosin (H&E) staining was used to observe the histopathological changes under a microscope. In addition, lung tissues were collected and detected for viral load using RT-PCR fluorescent TaqMan according to the instructions (Wuhan Grint Biology).

Serum samples were collected at 0, 1, 3, 4, 5, 6, 7, and 10 days after administration and then used for the detection of the Nb1-pFc concentration in the pigs’ blood using ELISA. Briefly, the 96-well ELISA plate was coated with PRRSV-N proteins (400 ng/well) and incubated overnight at 4°C. After being blocked with 200 µL 2.5% (wt/vol) non-fat dry milk, the plates were added with 50 µL testing serum samples and incubated for 1 h at RT. Next, the plates were added rabbit anti-camel polyclonal antibody (1:1,000) and, after incubation for 1 h, added HRP-conjugated goat anti-rabbit IgG (1:5,000).

### Expression and purification of different truncated and mutated PRRSV-N proteins

To identify the epitope recognized by the nanobody, different truncated and mutated PRRSV-N proteins were designed and expressed using the *Escherichia coli* (*E. coli*) system (24). The genes encoding truncated PRRSV-2 N proteins (amino acids 1–95, 30–124, 30–113, 1–102, and 1–109) and the recombinant PRRSV N protein with Myc tag were amplified using a pET28a-N plasmid as the template (24). In addition, the gene encoding complete PRRSV-1 N protein was also amplified using GZ11-G1 viral stock as the template (34). Next, the PCR products were purified and cloned into the pET-28a vector (Novagen). The sequences of the primers for PCR amplification are listed in Table 1. After sequencing, the positive plasmids were transformed into *E. coli* BL21 (DE3) (TransGen Biotech Co. Ltd.). Next, the proteins were expressed and purified as described previously (24).

After the antigenic domain was identified using the above-truncated PRRSV N proteins, alignments of amino acids between PRRSV-1 and −2 were determined by Lasergene 7.1 software. Then, based on the alignments, the four mutated PRRSV N proteins (N^3M^, N^S105A^, N^T108A^, and N^Q109A^) were designed and generated using overlap PCR site**-**directed mutagenesis followed by cloning into the pET-28a vector. The sequences of the primers used for PCR amplification are also listed in Table 1. These mutated proteins were also expressed and purified using the same methods as the complete PRRSV-2 N protein (24).

The complete, truncated, and mutated proteins were analyzed using SDS-PAGE and used as antigens for western blotting and ELISA to determine the interaction with the nanobody and identify the epitope recognized by the nanobody.

### ELISA

Direct ELISA was used to detect the interaction between truncated and mutated PRRSV-N protein and the nanobody, as previously described (24). Briefly, the 96-well ELISA plate was coated with different truncated and mutated PRRSV-N proteins (400 ng/well) and incubated overnight at 4°C. After being blocked with 200 µL 2.5% (wt/vol) non-fat dry milk in PBST and washed with PBST, the plates were added with 100 µL nanobody with HRP fusion protein and incubated for 1 h at RT. Next, the plates were washed again and added with 100 µL/well tetramethylbenzidine. Finally, 3 M H_2_SO_4_ (50 µL/well) was added to stop the colorimetric reaction, and the OD_450nm_ values were read using an automated ELISA plate reader (BioTek Instruments Inc.).

### Dot blot and peptide-based ELISA

In the dot blot assay, 2 µg of each peptide was spotted onto nitrocellulose membranes (AE99, Schleicher & Schuell Inc., Germany). After being dried, the membranes were blocked with 5% skimmed milk for 2 h at RT. Then, the membranes were incubated with primary antibodies for 1 h at RT. Finally, the membranes were visualized using an ECL substrate (Solarbio). In peptide-based ELISA, the 96-well ELISA plate was coated with peptides (4 µg/well) and incubated overnight at 4°C. After being blocked with 200 µL 2.5% (wt/vol) non-fat dry milk in PBST, the plates were added with 100 µL PRRSV-N-Nb1-HRP fusion proteins and incubated for 1 h at RT. Next, the plates were added with 100 µL/well tetramethylbenzidine. Finally, 3 M H_2_SO_4_ (50 µL/well) was added to stop the colorimetric reaction, and the OD_450nm_ values were read using an automated ELISA plate reader (BioTek Instruments Inc.). All peptides were synthesized by GenScript (Nanjing, China), and the purity of the synthetic peptides was equal to or greater than 95%.

### Reverse genetics-based mutagenesis of PRRSV

The mutated infection clones of PRRSV were constructed as described previously (26). Briefly, amino acid residues at positions S105, T108, and Q109 of PRRSV-2 N protein were mutated via site-directed mutagenesis using the primers listed in Table 1. After the PCR products were cloned into pBAC-rSD16 with *Asc* I and *Rsr* II enzymes (NEB, Ipswich, MA, USA), the different mutated infection clones of PRRSV were separately termed rSD16-N^S105A^, rSD16-N^T108A^, and rSD16-N^Q109A^. Plasmids of these mutated infectious clones were extracted using a Hispeed Plasmid Maxi Kit according to the manufacturer’s protocol (Qiagen, Hilden, Germany). To rescue viruses, rSD16-N^S105A^, rSD16-N^T108A^, rSD16-N^Q109A^, and wild-type rSD16 were transfected into MARC-145 cells using X-tremeGENE HP DNA Transfection Reagent (Roche Diagnostics GmbH). At 72 hpt, the cell culture supernatants were collected and serially passaged in MARC-145 cells up to three times. Then, the supernatant from the final passage was used to inoculate MARC-145 cells. At 48 hpi, IFA, western blotting, and TCID_50_ assays were performed to confirm the replication of the mutated viruses.

### Analysis of the nanobody with pFc fusion protein being involved in the stages of viral replication

As we know, the life cycle of PRRSV replication includes four stages: attachment, entry, genome replication, viral assembly, and release. The stages of attachment, entry, and genome replication were first determined. Briefly, the PAMs were treated with 40 µM Nb1-pFc or Nb53-pFc for 2 h. Then, cells were infected with 5 MOI PRRSV strain SD16 at 4°C for 1 h (attachment) or transferred to 37°C for 1 h (entry) or 8 h (genome replication). Then, the cells were collected to analyze virus attachment, entry, and genome replication by RT-qPCR as described above. Secondly, to evaluate virus release, the PAMs were infected with 5 MOI PRRSV strain SD16 at 4°C for 1 h and then transferred to 37°C. At 10 hpi, the cells were washed three times with PBS and added with 40 µM Nb1-pFc or Nb53-pFc for 2 h. The viral copies were detected in cells and supernatant by RT-qPCR, respectively. The viral assembly was analyzed as below.

### Analysis of the nanobody with pFc fusion protein blocking the self-interaction of PRRSV N protein

Previous studies documented that the main functions of PRRSV N protein in viral replication are to encapsulate the viral RNA genome and then to be involved in viral assembly (17). Additionally, the self-interaction of PRRSV-N protein to form a dimmer is important for encapsulating viral RNA genome and viral assembly (16). Then, the 3D structures of homology modeling for PRRSV N protein and the nanobody were first generated by the amino acid sequences submitted to the AlphaFold2 server (35). The docking model of interaction between PRRSV-N-Nb1 and PRRSV-N proteins was developed using the docking program on the server ClusPro (cluspro.bu.edu/home.php) (36). Interaction sites were analyzed using PyMOL (pymol.org/2/support.html). Prediction of two PRRSV-N proteins’ complex structures was also performed as described above. Based on the docking models of PRRSV N protein with the nanobody and self-interaction of PRRSV N protein, the inhibition of nanobody to self-interaction of PRRSV N protein was analyzed and predicted.

Secondly, a blocking ELISA was performed to verify the nanobody’s binding to PRRSV N protein, affecting self-binding. The 96-well ELISA plate was coated with PRRSV N protein or mutated PRRSV N proteins (N^3M^, N^S105A^, N^T108A^, and N^Q109A^) with His tag (10 µg/well) and incubated overnight at 4°C. After being blocked with 200 µL 2.5% (wt/vol) non-fat dry milk in PBST and washed with PBST, the plates were added with the different concentrations of Nb1-pFc and Nb53-pFc fusion protein (2, 1, and 0 µg/well or 10, 6, 4, 2, and 1 µg/well) and incubated for 1 h at RT. Next, the plates were washed again and added with different concentrations of PRRSV N protein with Myc tag (800, 400, and 200 ng/well or 800 ng/well). Subsequently, the primary antibodies, mouse anti-Myc mAb (1:2,000; ProteinTech Group Inc.), and the secondary antibody, HRP-conjugated goat anti-mouse IgG (1:5,000), were added and incubated for 1 h at RT. Then, the TMB was added for color reaction. Finally, 3 M H_2_SO_4_ (50 µL/well) was added to stop the colorimetric reaction, and the OD_450nm_ values were read using an automated ELISA plate reader.

### Transmission electron microscopy

To analyze the expression of PRRSV N protein and the assembly of wild-type and mutated PRRSV, IFA and transmission electron microscopy (TEM) were performed as described previously (37) with the following modifications. Briefly, each 2 µg rSD16-N^S105A^ and rSD16 plasmids were transfected into HEK293T cells using X-tremeGENE HP DNA Transfection Reagent (Roche, Basel, Switzerland). At 48 hpt, the cell supernatants were collected for infection MARC-145 cells, and the transfected HEK294T cells were fixed for IFA. Then, 100 µL of cell supernatants were added to MARC-145 cells. At 12 hpi, the cells were collected, centrifuged at 300 × *g* at RT for 10 min, washed with PBS, and centrifuged again. The treated cells were fixed with 2.5% glutaraldehyde (Sigma-Aldrich; Merck KGaA) at 4°C for 2 h. Samples were negatively stained using 2% phosphotungstic acids at RT for 2 min. Finally, the cuprum grids were observed under a TEM, and images were taken (Hitachi Ltd., HT7800). Images were taken at an 8.0 k × magnification.

### Statistical analysis

Statistical analysis was performed using GraphPad Prism version 9.0. Statistical significance between the two groups was analyzed using unpaired Student’s *t*-test, and differences between three or more groups were compared with a control group using a one-way analysis of variance (ANOVA). Asterisks indicate the statistical significance: NS, no significance; *P < 0.05, **P < 0.01, and ***(*P* < 0.001). *P* < 0.05 was considered to indicate a statistically significant difference.

## RESULTS

### Intracellular expression of PRRSV-N-Nb1 significantly inhibits PRRSV replication in MARC-145 cells

**Fig 1:**
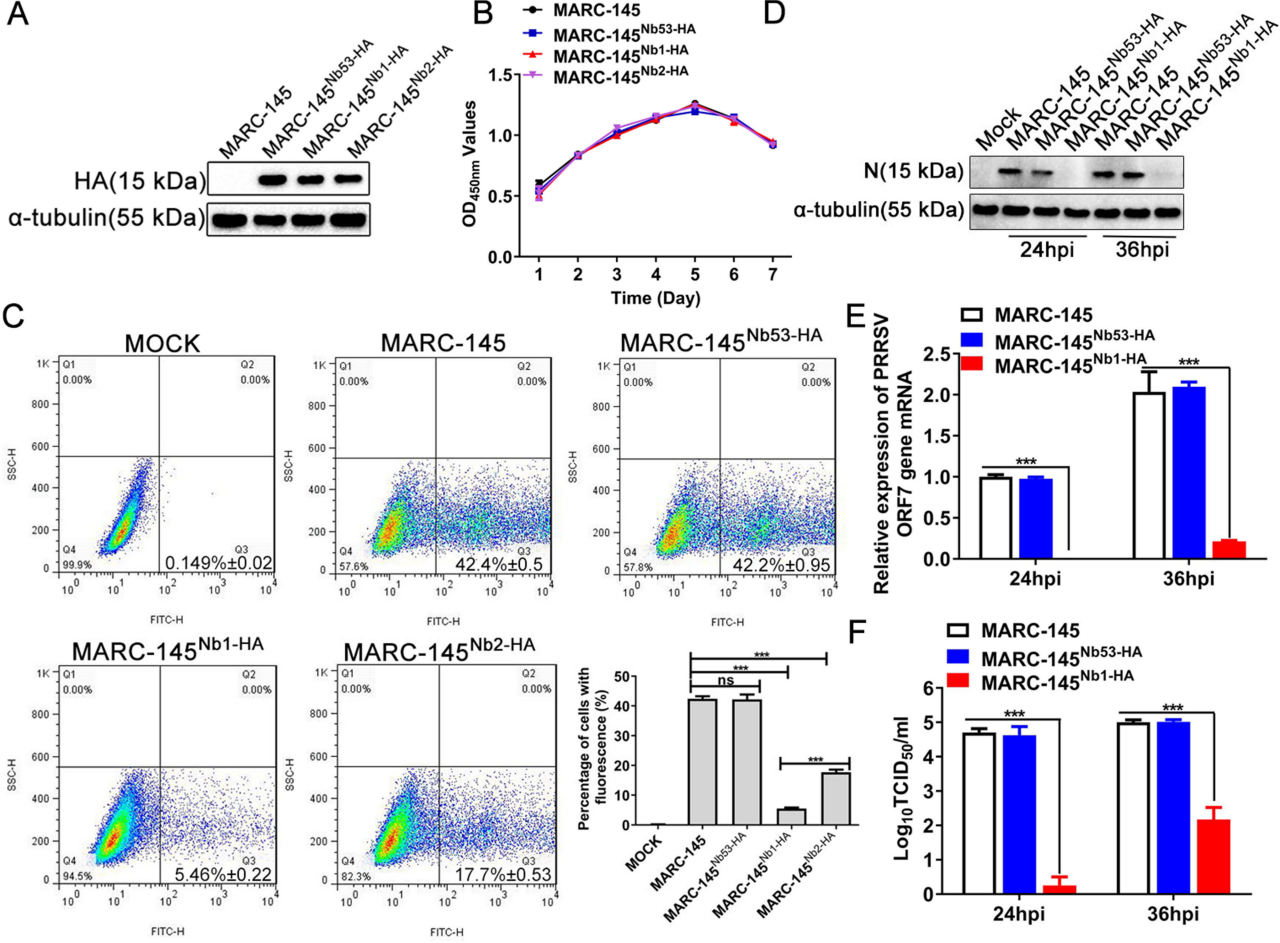
Intracellular expression of PRRSV-N-Nb1 and -Nb2 inhibits PRRSV proliferation in MARC-145 cells. (**A**) Establishment of MARC-145 cell lines stably expressing PRRSV-N-Nb1, -Nb2, or -Nb53. Expression of nanobodies in the MARC-145 cell lines was determined by western blotting using an anti-HA mAb as the primary antibody. (**B**) Cell growth curves of MARC-145, MARC-145^Nb1-HA^, MARC-145^Nb2-HA^, and MARC-145^Nb53-HA^ cells. (**C**) The positive proportion of MARC-145, MARC-145^Nb1-HA^, MARC-145^Nb2-HA^, and MARC-145^Nb53-HA^ cells infected with GFP-PRRSV with an 0.1 MOI based on flow cytometry analysis. The positive proportion was measured in living cells. (**D**) Western blotting analysis of N protein in MARC-145^Nb1-HA^ inoculated with PRRSV SD16 strain. (**E**) Reverse transcription-quantitative PCR analysis of ORF7 mRNA in MARC-145^Nb1-HA^ inoculated with PRRSV SD16 strain. (**F**) TCID_50_ of the progeny virus in the supernatant of MARC-145^Nb1-HA^ inoculated with the PRRSV SD16 strain. Data are expressed as the mean ± standard deviation of three repeats. Differences between groups were compared using unpaired Student’s *t*-test. ^***^P < 0.001 vs. control cells. ns, not significant; PRRSV, porcine reproductive and respiratory syndrome virus; N, nucleocapsid; mAb, monoclonal antibody; MOI, multiplicity of infection.

In a previous study, two anti-PRRSV-N protein nanobodies (PRRSV-N-Nb1 and -Nb2) were screened (24). In the present study, the ability of two nanobodies to inhibit PRRSV replication in MARC-145 cells was first analyzed. Three recombinant MARC-145 cell lines intracellularly expressing PRRSV-N-Nb1, -Nb2, and Nb53 (negative control) were constructed. Western blotting analysis showed that the bands of the expected size of 15 kDa were detected in the three recombinant cell lines but not in wild-type MARC-145 cells (Fig. 1A), indicating that the recombinant cell lines were successfully established. The three cell lines were separately termed MARC-145^Nb1-HA^, MARC-145^Nb2-HA^, and MARC-145^Nb53-HA^. Then, CCK-8 assays were performed to analyze the growth of the three recombinant and wild-type cell lines. The results showed that there were no significant differences between the three recombinant and wild-type MARC-145 cell lines (Fig. 1B). Subsequently, after the cells were infected with GFP-PRRSV strains at 0.1 MOI for 36 h, FCM results showed that the positive rate of GFP fluorescence in wild-type MARC-145 cells was 42.4% ± 0.5, MARC-145^Nb53-HA^ was 42.2% ± 0.95, MARC-145^Nb1-HA^ was 5.46% ± 0.22, and MARC-145^Nb2-HA^ was 17.7% ± 0.53 (Fig. 1C), indicating that PRRSV-N-Nb1 and -Nb2 can inhibit PRRSV replication in MARC-145 cells, and PRRSV-N-Nb1 exhibited the greatest effect. Thus, PRRSV-N-Nb1 was selected for subsequent experiments.

To further determine whether the wild-type PRRSV isolate can also be inhibited by PRRSV-N-Nb1, MARC-145, MARC-145^Nb53-HA^, and MARC-145^Nb1-HA^ cells were infected with a wild-type PRRSV strain SD16 at an MOI of 0.1, and the cells and supernatant were collected at 24 and 36 hpi. The results of RT-qPCR and western blotting showed that compared with MARC-145 and MARC-145^Nb53-HA^ cells, there were significantly decreased levels of PRRSV ORF7 mRNA and N protein in MARC-145^Nb1-HA^ cells at 24 and 36 hpi (Fig. 1D and E). In addition, the TCID_50_ of progeny virus also showed that the amount of PRRSV in the supernatant of MARC-145^Nb1-HA^ cells significantly decreased at 24 hpi and 36 hpi and decreased by ~3 logs at 36 hpi (Fig. 1F).

### Production and characterization of Nb1-pFc fusion protein

**Fig 2:**
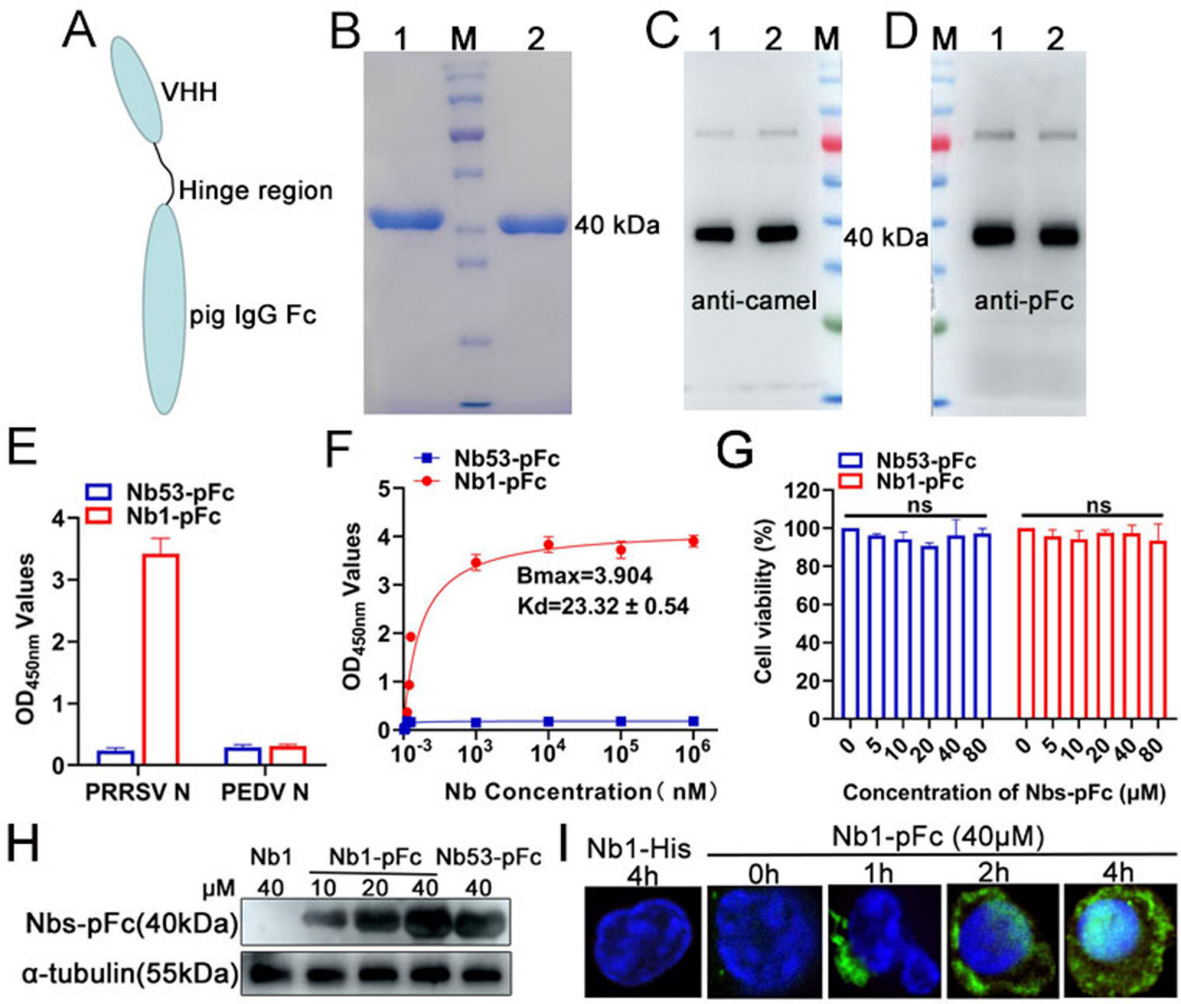
Production and characterization of Nb1-pFc fusion protein. (**A**) Structural model of Nb1-pFc fusion proteins. (**B**) SDS-PAGE analysis of the Nb1-pFc fusion protein. Antigenic analysis of the Nb1-pFc fusion proteins by western blotting separately using murine anti-camel serum (**C**) and HRP-conjugated goat anti-swine IgG (**D**) as first antibodies. M: molecular weight markers; Lane 1: Nb53-pFc fusion protein; Lane 2: Nb1-pFc fusion protein. (**E**) Nb1-pFc specifically reacted with PRRSV-2 N protein detected by the indirect ELISA. The recombinant PEDV N protein and Nb53-pFc were used as a negative control. (**F**) The affinity of the Nb1-pFc fusion protein binding to PRRSV-2 N protein. (**G**) Cytotoxicity of Nb1-pFc fusion proteins using detection of CCK8 kits. Western blotting (**H**) and IFA (**I**) analysis of Nb1-pFc entry into PAMs at different concentrations or times. Data are expressed as the mean ± standard deviation of three repeats. *P-*values were calculated using one-way ANOVA; ns, not significant.

The PRRSV-N-Nb1 was selected and expressed with a pFc tag using HEK293T cells based on the above results. The pFc was used as the delivering tag to help the nanobodies enter the PAMs (Fig. 2A). The nanobodies with pFc fusion proteins were then used to analyze the effects of inhibiting PRRSV replication in PAMs. The negative Nb53 was also a negative control and expressed with a pFc tag. SDS-PAGE analysis showed that the two fusion proteins of nanobodies (Nb1-pFc and Nb53-pFc) with the expected size of 40 kDa were successfully expressed in the HEK293T and purified with protein G resin (GenScript, China; the purity was >95%) (Fig. 2B). Additionally, the Nb1-pFc and Nb53-pFc could be detected by rabbit anti-camel polyclonal antibody and HRP-conjugated goat anti-swine IgG, suggesting that they still have good immunological activity of nanobody and pFc (Fig. 2C and D). The specificity of Nb1-pFc to PRRSV-N protein was also assessed by indirect ELISA, which showed that Nb1-pFc only binds to PRRSV N protein and not to PEDV N protein (Fig. 2E). In addition, the binding affinity of Nb1-pFc was determined, and as shown in Fig. 2F, the affinity constant of Nb1-pFc to the N protein was 23.32 ± 0.54 HAU mL. Subsequently, the CCK-8 assays were performed to analyze the toxicity of Nb1- and Nb53-pFc in the PAMs. The results showed that the two fusion proteins are not cytotoxic at concentrations lower than 80 µM (Fig. 2G).

To verify the ability of Nb1-pFc to enter PAM, the cells treated with Nb1-pFc or Nb53-pFc were examined using western blotting and indirect immunofluorescence assay (IFA). The western blotting results showed that Nb1-pFc and Nb53-pFc were detected at various concentrations in PAMs (Fig. 2H). In contrast, Nb1-His without pFc was not detected (Fig. 2H). Nb1-pFc was incubated at 40 µM for different periods (1, 2, and 4 h) in PAMs, and the Nb1-pFc was then detected using IFA. The data showed that increasing amounts of Nb1-pFc were delivered into the PAMs at times ranging from 0 to 4 h. In addition, Nb1-pFc is distributed in both cytoplasm and nucleus but mainly in cytoplasm (Fig. 2I). In conclusion, the Nb1-pFc can enter into the PAMs.

### Nb1-pFc significantly inhibits PRRSV replication in the PAMs

**Fig 3:**
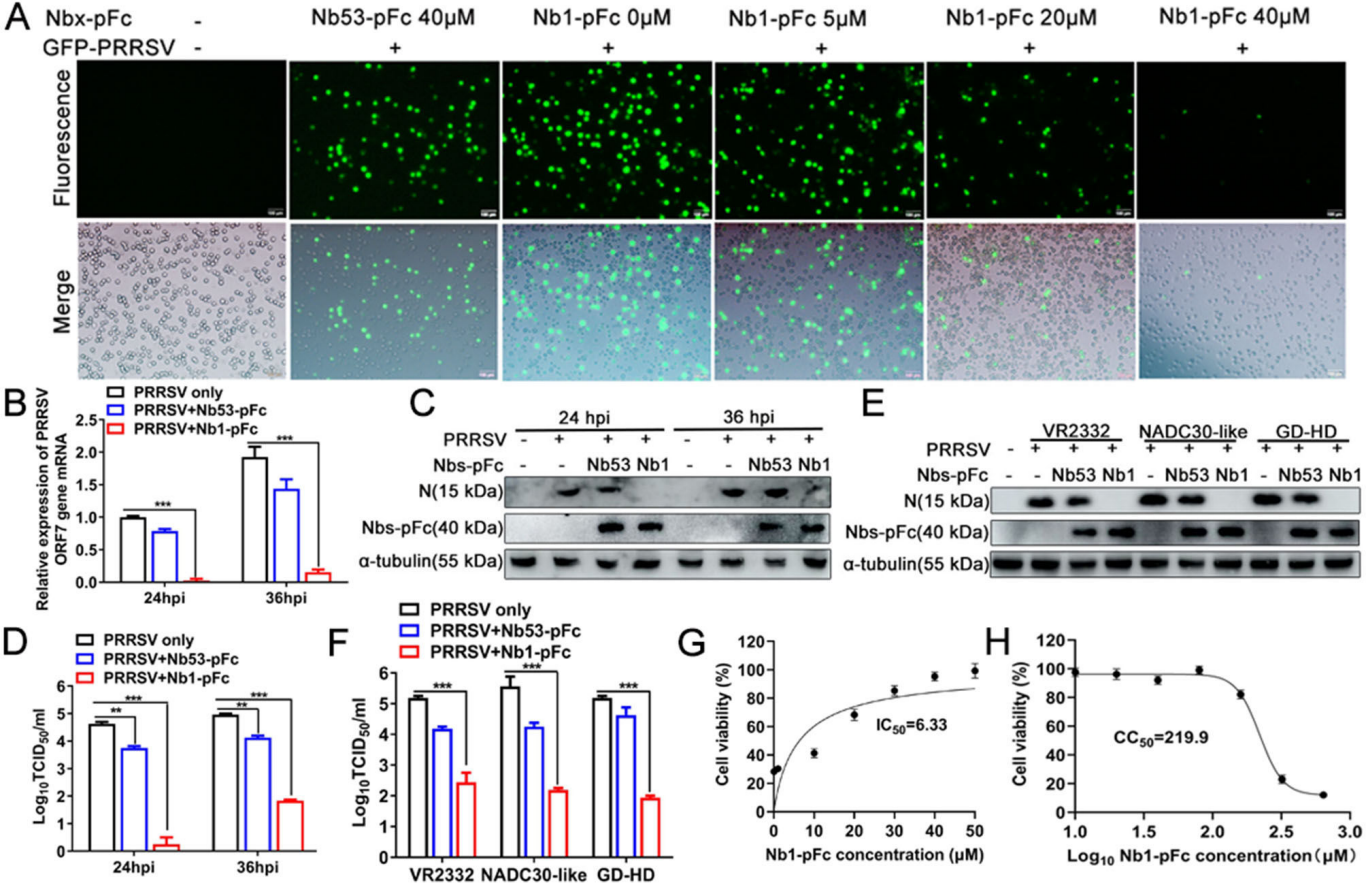
Nb1-pFc significantly inhibits PRRSV replication in PAMs. The PAMs were treated with different concentrations of Nb1-pFc and then infected with PRRSV isolates with 0.01 MOI. (**A**) The positive cells were observed using fluorescence microscopy. (**B**) Reverse transcription-quantitative PCR analysis of ORF7 mRNA after the cells were inoculated with PRRSV SD16 strain at 24 hpi and 36 hpi and (**C**) Nb1-pFc and PRRSV-2 N proteins were detected using western blotting. (**D**) TCID_50_ of the progeny virus in the supernatant. (**E**) Western blotting analysis of N protein in PAMs inoculated with the PRRSV VR2332, NADC30-like, and GD-HD strains. (**F**) TCID_50_ of the progeny virus in the supernatant of PAMs inoculated with PRRSV GD-HD, NADC30-like, and VR2332 strains. IC50 (**G**) and CC50 (**H**) of the Nb1-pFc were determined by CCK-8 assay.

To evaluate the antiviral activity of Nb1-pFc, the PAMs were infected with GFP-PRRSV strains at 0.01 MOI for 36 h. After 36 hpi, the green positive cells were observed under a fluorescence microscope. Nb1-pFc significantly inhibited PRRSV replication with dose-dependent results, and the inhibition rate was >90% when the concentration of Nb1-pFc was 40 µM (Fig. 3A).

To further determine that the Nb1-pFc can also inhibit wild-type PRRSV isolate replication, the PAMs were first incubated with 40 µM Nb1-pFc or Nb53-pFc for 2 h. Then, the cells were infected with PRRSV strain SD16 at an MOI of 0.01, and the cells and supernatant were collected at 24 hpi and 36 hpi, respectively. The results of RT-qPCR showed that compared with only PRRSV and Nb53-pFc, there were significantly decreased levels of PRRSV ORF7 mRNA in Nb1-pFc at 24 and 36 hpi (Fig. 3B). Furthermore, the PRRSV N protein was undetectable at 24 and 36 hpi after the cells were treated with Nb1-pFc by western blotting (Fig. 3C). In addition, the TCID_50_ of progeny virus also showed that the amount of PRRSV in the supernatant of treated Nb1-pFc cells significantly decreased at 24 hpi and 36 hpi and decreased by 3 logs at 36 hpi (Fig. 3D).

To further explore whether Nb1-pFc can inhibit replication of different PRRSV-2 strains, three isolates, including VR2332, NADC30-like, and GD-HD, were used to infect PAMs at an MOI of 0.1 after the cells were treated with Nb1-pFc. At 36 hpi, the results showed that the levels of PRRSV-N protein by western blotting were undetectable, and the TCID_50_ of progeny virus was significantly decreased (Fig. 3E and F), indicating that the Nb1-pFc fusion protein can significantly inhibit replication of different PRRSV-2 strains in the PAMs. To determine whether the anti-PRRSV activity of Nb1-pFc can be attributed to its cytotoxic effect, the IC50 and CC50 values were determined in PAMs. The IC50 and CC50 values of Nb1-pFc fusion protein were 6.33 µM and 219.9 µM, respectively (Fig. 3G and H).

### Nb1-pFc treatment alleviates pulmonary pathological lesions of piglets infected with HP-PRRSV and inhibits viral replication *in vivo*

**Fig 4:**
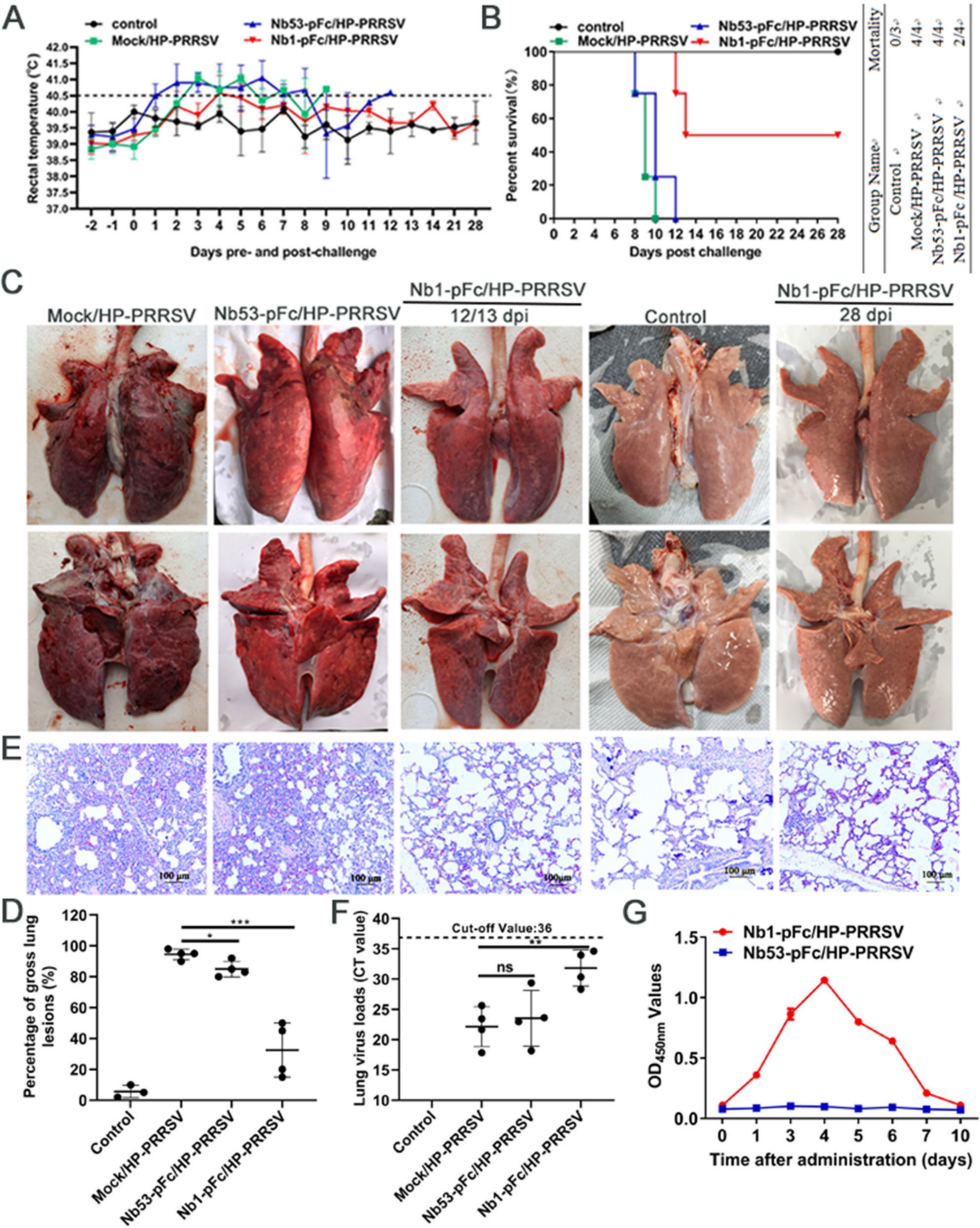
Nb1-pFc can inhibit HP-PRRSV JX1 strain replication in the piglets and reduce lung lesions. (**A**) Rectal temperature was recorded daily for surviving animals of all groups. (**B**) The mortality and survival curves of piglets in each group are shown (*n* = 4/3). (**C**) Gross lesions of a pig’s lungs. For each group, representative images were captured immediately after piglets were autopsied at death or 28 dpi. (**D**) The scores of gross lung lesions in each piglet were based on the percentage of lung area affected using a scoring (100-point) system. Each lung lobe was assigned several points (100 points in total). (**E**) The microscopic lung lesions. Tissue samples from the lungs were fixed, embedded, and stained with H&E to facilitate the observation of micro-pathological changes. Representative images were captured, and the scale bar represents a length of 100 µm. (**F**) Viral copies of lung tissue in each pig were detected using RT-PCR fluorescent TaqMan. (**G**) The half-life of Nb1-pFc in pigs. Differences between groups were compared using unpaired Student’s *t*-test. *P < 0.05, **P < 0.01, and ***P < 0.001; ns, not significant.

To further analyze the anti-PRRSV activity of Nb1-pFc *in vivo*, piglets infected with HP-PRRSV (JXA1) were treated with Nb1-pFc. Then, daily rectal temperatures, death of pigs, and lung pathological injury were recorded in all piglets. On the second day after the challenge, the rectal temperature of the Mock/HP-PRRSV and Nb53-pFc/HP-PRRSV groups rose to more than 40.5°C, the groups sustained high fever for 7 days (Fig. 4A), and they groups successively died. However, the Nb1-pFc treatment group developed a high fever on the fourth day after the challenge, and the rectal temperature returned to normal at 6 dpi (Fig. 4A). The rectal temperature was normal in the control group during the experiment period.

All piglets in the Mock/HP-PRRSV and Nb53-pFc/HP-PRRSV groups died at 12 dpi (Fig. 4B). One piglet in the Mock/HP-PRRSV group died at 8 and 10 dpi, respectively, and two died at nine dpi. In the Nb53-pFc/HP-PRRSV group, one piglet died at 8 and 12 dpi, respectively, and two died at 10 dpi. In the Nb1-pFc/HP-PRRSV group, one piglet died at 12 and 13 dpi, respectively, and two pigs survived during the study (Fig. 4B).

Necropsies and gross lung lesion examinations were performed on dead and surviving pigs during the experiment. The pig lung tissue presented severe pulmonary edema, hemorrhage, and consolidation in the Mock/HP-PRRSV and Nb53-pFc/HP-PRRSV groups, with phenotypic similarities (Fig. 4C). Two piglets died at 12 and 13 dpi in the Nb1-pFc/HP-PRRSV group which also presented pulmonary edema and hemorrhage but less severe than the piglets in the Mock/ and Nb53-pFc/HP-PRRSV groups (Fig. 4C). Additionally, the pigs were euthanized at 28 dpi from the Nb1-pFc/HP-PRRSV, and no-challenge groups were also compared and did not show any gross lung lesions (Fig. 4C), suggesting that the lesions of two surviving pigs in the Nb1-pFc/HP-PRRSV group were probably recovered. To quantify better these pathological changes, a lung gross lesion score system was also applied as previously described (32). The gross lung lesion scores of the piglets in the Mock/HP-PRRSV (94.5 ± 3.3) and Nb53-pFc/HP-PRRSV groups (85 ± 5.1) were significantly higher than those of two piglets that died at 12 or 13 dpi in the Nb1-pFc/HP-PRRSV group (47.5 ± 3.5) (Fig. 4D). The scores of all the four piglets in the Nb1-pFc/HP-PRRSV group were 32.5 ± 17.5 (Fig. 4D). The pigs in the no-challenge control group exhibit gross lesions in the lungs.

The microscopic lesions of the lungs were also observed. Compared with the no-challenge control group, marked interstitial pneumonia, including diffuse increasing of inflammatory infiltrates (mononuclear) in alveolar walls, thickening of alveolar walls, and diffuse hemorrhages, was observed in the pigs from the Mock/HP-PRRSV group. There were hyaline thrombi in the microvessel. Similar to the Mock/HP-PRRSV group, a diffuse increase of inflammatory infiltrates (mononuclear) in alveolar walls and diffuse hemorrhages was also observed in the Nb53-pFc/HP-PRRSV group and with hyaline thrombi in the microvessel. The dead pigs at 12 and 13 dpi in the Nb1-pFc/HP-PRRSV group also showed inflammatory infiltration, but it was lighter than the ones from Mock/HP-PRRSV and Nb53-pFc/HP-PRRSV groups (Fig. 4E). In contrast, the lungs of survived piglets from the Nb1-pFc-treated group had only slight pathological changes. Only diffuse hemorrhages were observed. There were no marked alveolar wall thickening and thrombosis (Fig. 4E).

In addition, the viral loads in the lungs were tested further to evaluate the degree of protection in the Nb1-pFc-treated group. Postmortem examinations and lung tissue collection were performed on dead and surviving pigs at 28 dpi, and the viral load of lung tissue in each pig was detected by RT-PCR fluorescent TaqMan. The results showed that the viral RNA levels in the lungs of the Nb1-pFc-treated group were significantly reduced 8 CT values compared with those in the Mock/HP-PRRSV and Nb53-pFc/HP-PRRSV groups (*P* < 0.05, Fig. 4F). And the viral RNA levels in the lungs of two piglets that died at 12 or 13 dpi from the Nb1-pFc-treated group were reduced 6 CT values compared with the other two groups (Fig. 4F). Finally, the Nb1-pFc concentration in the pigs’ blood was also detected using ELISA, and the ELISA results showed that the half-life of Nb1-pFc was about 4 days (Fig. 4G). In conclusion, the treatment of Nb1-pFc can effectively reduce lung injury caused by infection of HP-PRRSV JXA1 strain and inhibit viral replication in pigs.

### Interaction between Nb1-pFc and N protein in PRRSV-infected cells

**Fig 5:**
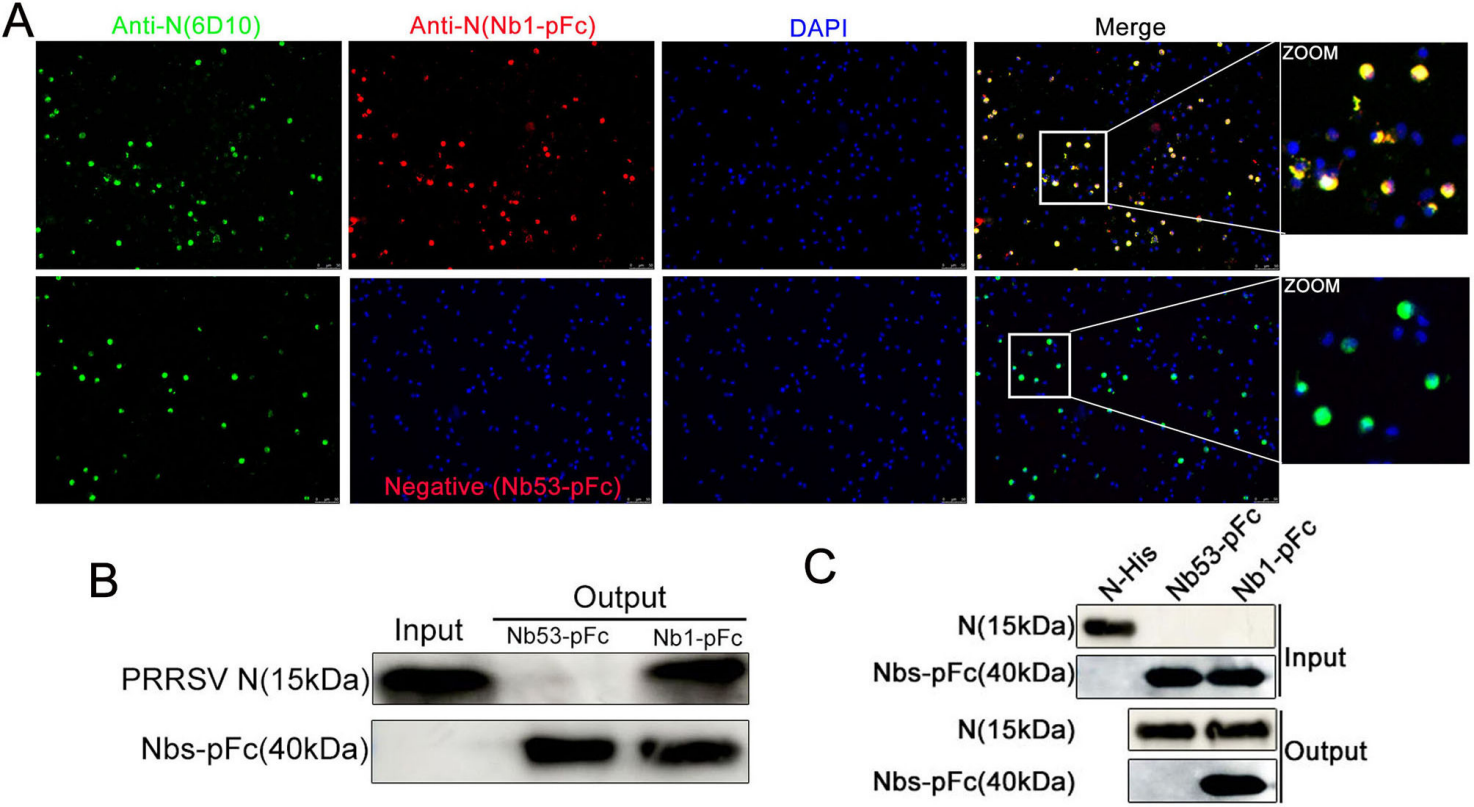
Interaction between Nb1-pFc and N protein in PRRSV-infected PAM cells. (**A**) IFA detection of Nb1-pFc binding to PRRSV-N protein using fluorescence microscopy. Magnification, 20×. PRRSV N protein fluoresced green, Nb1 and Nb53-pFc fluoresced red, and the nuclei fluoresced blue. (**B**) PRRSV N was pulled down using the Nb1-pFc as the bait by a pull-down assay in PRRSV-infected PAMs. (**C**) Nb1-pFc was pulled down using the N protein as the bait under the exogenous expression of the two proteins. PRRSV, porcine reproductive and respiratory syndrome virus; N, nucleocapsid.

To verify the binding of Nb1-pFc to PRRSV N protein in the PAMs, the cells were first infected with 0.5 MOI PRRSV for 24 h. Then, the IFA using Nb1-pFc as the primary antibodies was performed. The mAb 6D10 against PRRSV N protein was used as a positive control. The results showed that the Nb1-pFc can still bind to the N protein in the PRRSV-infected cells (Fig. 5A). In addition, the pull-down assay with Nb1-pFc as the bait protein also showed that the N protein in PRRSV-infected PAM cells could be pulled down by the Nb1-pFc and not by the Nb53-pFc (Fig. 5B). To further confirm the interaction between Nb1-pFc and N protein, a co-immunoprecipitation (co-IP) assay was performed by exogenous expression of the two proteins. As shown in Fig. 5C, the N protein as the bait protein also showed that the Nb1-pFc protein could be pulled down, and the Nb53-pFc was not. These results indicated that the Nb1-pFc inhibited PRRSV replication by binding to the N protein.

### Identification of the epitope recognized by PRRSV-N-Nb1

**Fig 6:**
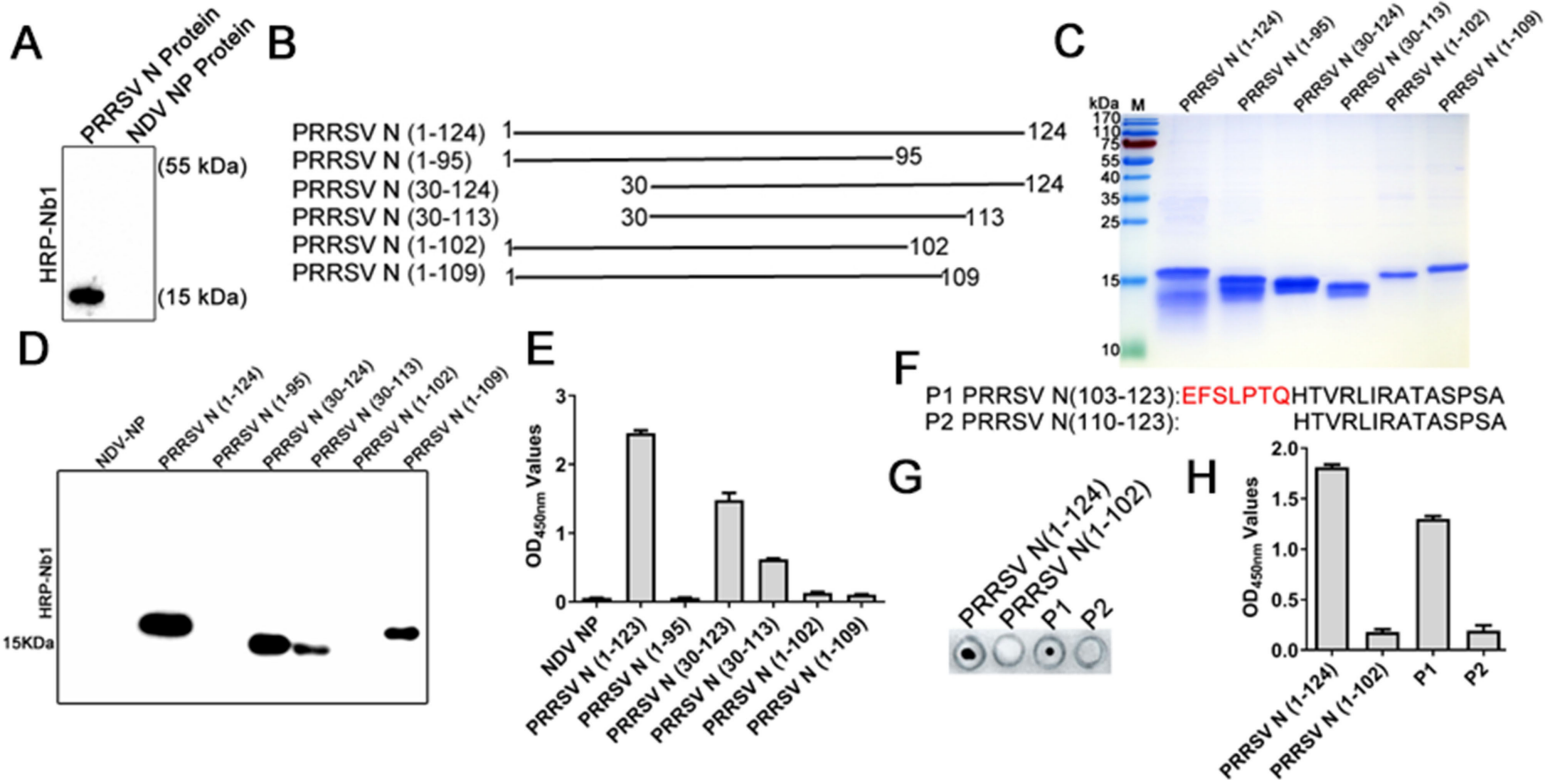
PRRSV-N-Nb1 binds to the amino acid 103–109 region of PRRSV N protein. (**A**) PRRSV-N-Nb1 reacting with PRRSV N protein expressed by *E. coli* as determined using western blotting. (**B**) Schematic diagram of different truncated PRRSV N proteins. (**C**) Expression and purification of different truncated PRRSV N proteins using the *E. coli* system. (**D**) The interaction of PRRSV-N-Nb1 with the different truncated PRRSV N proteins was determined using western blotting. (**E**) Analysis of interaction between PRRSV-N-Nb1 and different truncated PRRSV N proteins using ELISA. (**F**) Schematic diagram of the synthetic polypeptides. A dot blot (**G**) and peptide-based ELISA (**H**) were used to analyze the reactivity of PRRSV N-Nb1 with the synthetic peptides. Complete PRRSV N (1–124) protein as the positive control and truncated N (1-102) protein as the negative control. PRRSV, porcine reproductive and respiratory syndrome virus; N, nucleocapsid.

Using the recombinant PRRSV-N protein as the antigen, western blotting analysis showed that PRRSV-N-Nb1 recognized the linear epitope of PRRSV-N protein (Fig. 6A). However, it could not react with the NDV-NP protein expressed using the same system as the PRRSV-N protein (Fig. 6A). Subsequently, to precisely determine the epitope recognized by PRRSV-N-Nb1, different truncated PRRSV-N proteins were designed and expressed (Fig. 6B). SDS-PAGE analysis showed that these fragments were successfully expressed with the expected sizes using the prokaryotic expression system and purified with Ni-resins (Fig. 6C). Using these fragments as antigens, western blotting results showed that PRRSV-N-Nb1 reacted with fragments spanning amino acids 30–124, 30–113, and 1–109 but not 1–95 and 1–102, suggesting that the epitope was located within amino acids 103–109 (Fig. 6D). The ELISA also provided mostly consistent results, except that the amino acid 1–109 fragments showed weak binding (Fig. 6E), which is probably because this protein folded and wraps the recognition epitope. To further determine the binding regions of Nb1, two polypeptides (P1 and P2) were synthesized (Fig. 6F). Dot blot and peptide-based ELISA showed that Nb1 reacted with P1 polypeptide but not with P2, which was consistent with the results of western blotting and ELISA (Fig. 6G and H). These results suggested that the epitope recognized by PRRSV-N-Nb1 was located within the region of amino acids 103–109.

**Fig 7:**
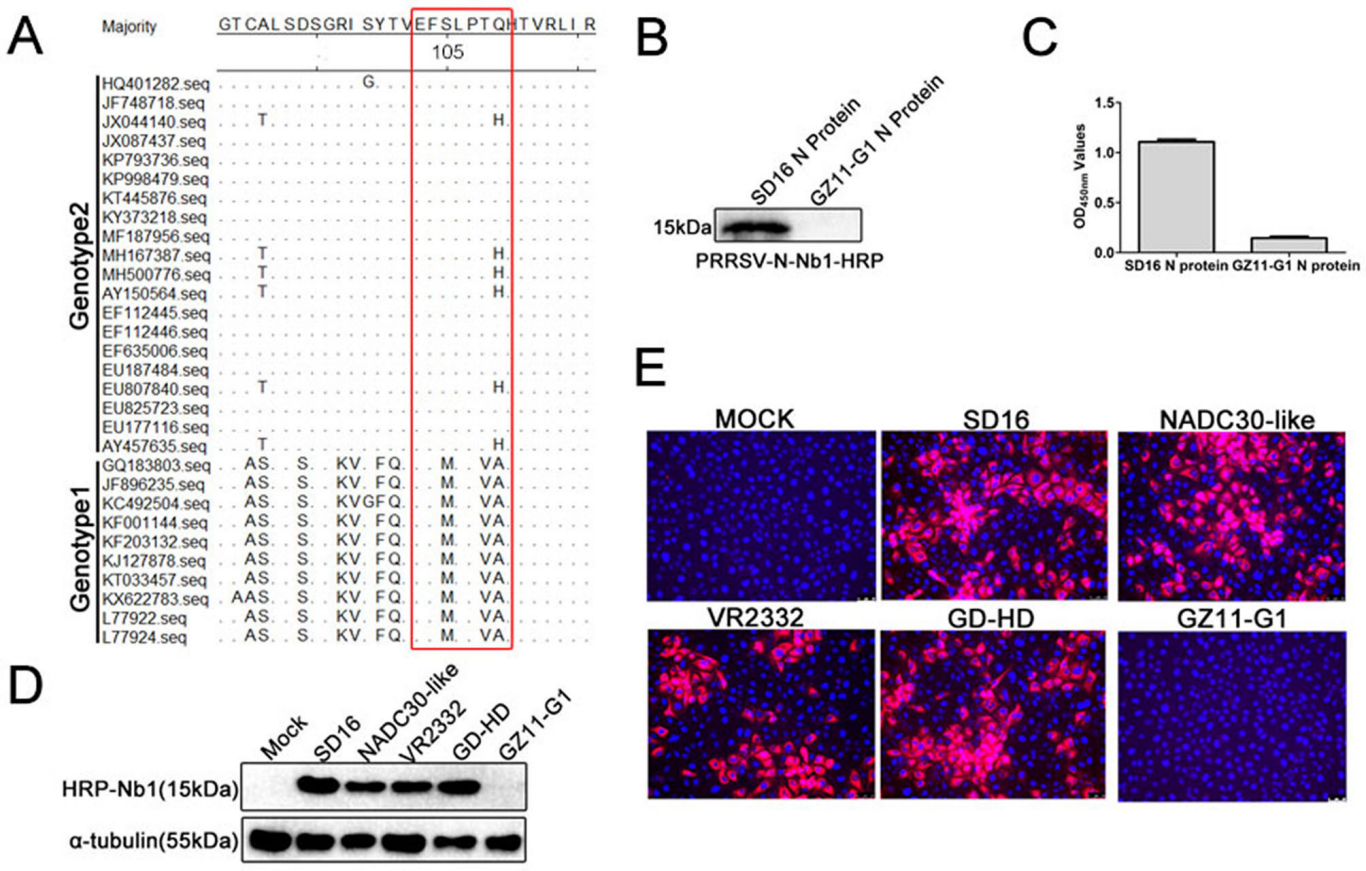
Fine mapping of the epitopes in PRRSV N protein recognized by PRRSV-N-Nb1. (**A**) Amino acid sequence alignments of amino acids 103–109 in the N protein of different PRRSV-1 and −2 isolates. The interaction between PRRSV-N-Nb1 and PRRSV-1 N protein was analyzed by western blotting (**B**) and ELISA (**C**). Analysis of PRRSV-N-Nb1 binding to the N proteins of different PRRSV isolates using western blotting (**D**) and immunofluorescence (**E**). NADC30-like, VR2332, and GD-HD isolates belong to PRRSV-2, and GZ11-G1 isolate is PRRSV-1. PRRSV, porcine reproductive and respiratory syndrome virus; N, nucleocapsid.

To further analyze the amino acid conservation of the epitope, the amino acid 103–109 regions of the N proteins from different PRRSV strains were aligned. Sequence alignments showed that the epitope was conserved among the same-genotype PRRSV strains (Fig. 7A). Only motif 109 was different among different PRRSV-2 strains (Fig. 7A). However, between PRRSV-1 and −2 strains, the sites of amino acids 105, 108, and 109 were different. The amino acids of PRRSV-2 were S105, T108, Q109, or H109 and PRRSV-1 were M105, V108, and A109 (Fig. 7A). Based on the alignments, PRRSV-1 N protein was successfully expressed, purified, and used as an antigen to react with PRRSV-N-Nb1. SDS-PAGE analysis showed that PRRSV-1 N protein was successfully expressed with the expected size using the same prokaryotic expression system as PRRSV-2 N protein (data not shown). Western blotting and ELISA showed that PRRSV-N-Nb1 could not react with PRRSV-1 N protein (Fig. 7B and C). To further confirm that PRRSV-N-Nb1 only binds to PRRSV-2 N proteins and not to PRRSV-1 ones, MARC-145 cells were infected with different strains of PRRSV-2 (SD16, NADC30-like, VR2232, and GD-HD) and of PRRSV-1 (GZ11-G1). Western blotting and IFA showed that PRRSV-N-Nb1 could react with PRRSV-2 N protein but not PRRSV-1 (Fig. 7D and E). Taken together, these results indicated that the 4 sites, S105, T108, Q109, or H109, may be important for PRRSV-N-Nb1 interaction with PRRSV-2 N protein.

### Motif S105 is the key amino acid for PRRSV-N-Nb1 interaction with PRRSV N protein

**Fig 8:**
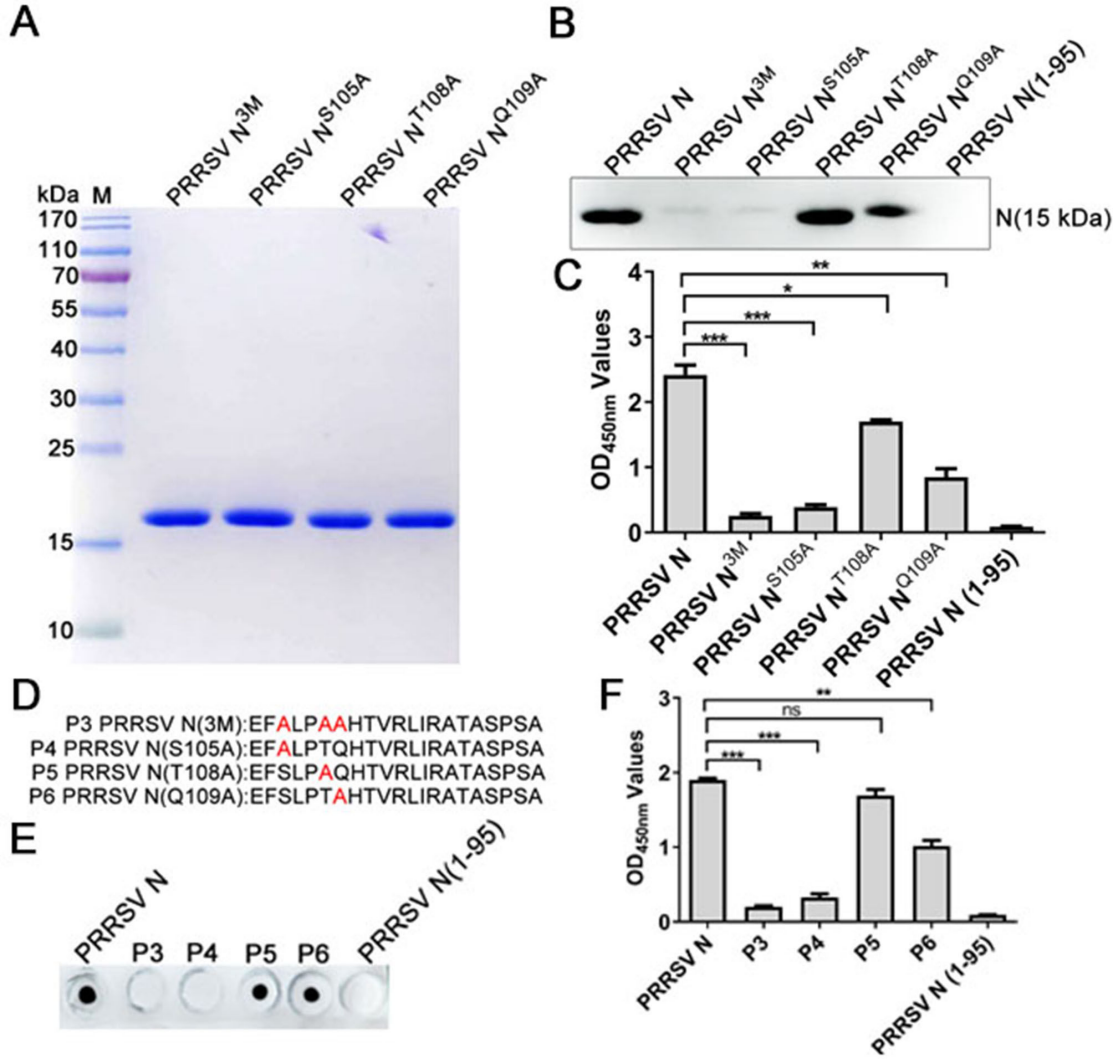
S105 is the key motif for PRRSV-N-Nb1 interaction with PRRSV-2 N protein. (**A**) Expression and purification of different mutated PRRSV-2 N proteins by SDS-PAGE analysis. (**B and C**) Analysis of the interaction between PRRSV-N-Nb1 and different mutated PRRSV-2 N proteins using western blotting (**B**) and ELISA (**C**). (**D**) Schematic diagram of the synthetic polypeptides. A dot blot (**E**) and peptide-based ELISA (**F**) analyze the reactivity of PRRSV-N-Nb1 with the synthetic polypeptides. Complete PRRSV-2 N protein as the positive control and truncated PRRSV-2 N (1-95) protein as the negative control. PRRSV, porcine reproductive and respiratory syndrome virus; N, nucleocapsid.

To further precisely define the key amino acid for PRRSV-N-Nb1 binding to PRRSV-2 N protein, three S105, T108, and Q109 sites were mutated into alanine. Then, all three singly mutated and one with all three site-mutated recombinant PRRSV-N proteins were expressed and purified. SDS-PAGE analysis showed that the four mutated proteins were successfully expressed with expected sizes and were termed PRRSV-N^3M^, PRRSV-N^S105A^, PRRSV-N^T108A^, and PRRSV-N^Q109A^ (Fig. 8A). Using the four mutated proteins as antigens, western blotting and ELISA showed that PRRSV-N^3M^ and PRRSV-N^S105A^ proteins did not react with PRRSV-N-Nb1 (Fig. 8B and C). Compared with wild-type PRRSV-2 N protein, the binding abilities of PRRSV-N^Q109A^ and PRRSV-N^T108A^ with PRRSV-N-Nb1 were weaker (Fig. 8B and C). To clarify the key binding sites of Nb1, systematic mutation peptides (P3–P6) were synthesized and evaluated by dot blot and peptide-based ELISA (Fig. 8D). The results showed that Nb1 reacted with P5 and P6 polypeptide but not with P3 and P4, which was consistent with western blotting and ELISA (Fig. 8E and F). These results indicated that S105 is the key motif for PRRSV-N-Nb1 interaction with PRRSV-2 N protein.

### Motif S105 is indispensable for the rescue of PRRSV

**Fig 9:**
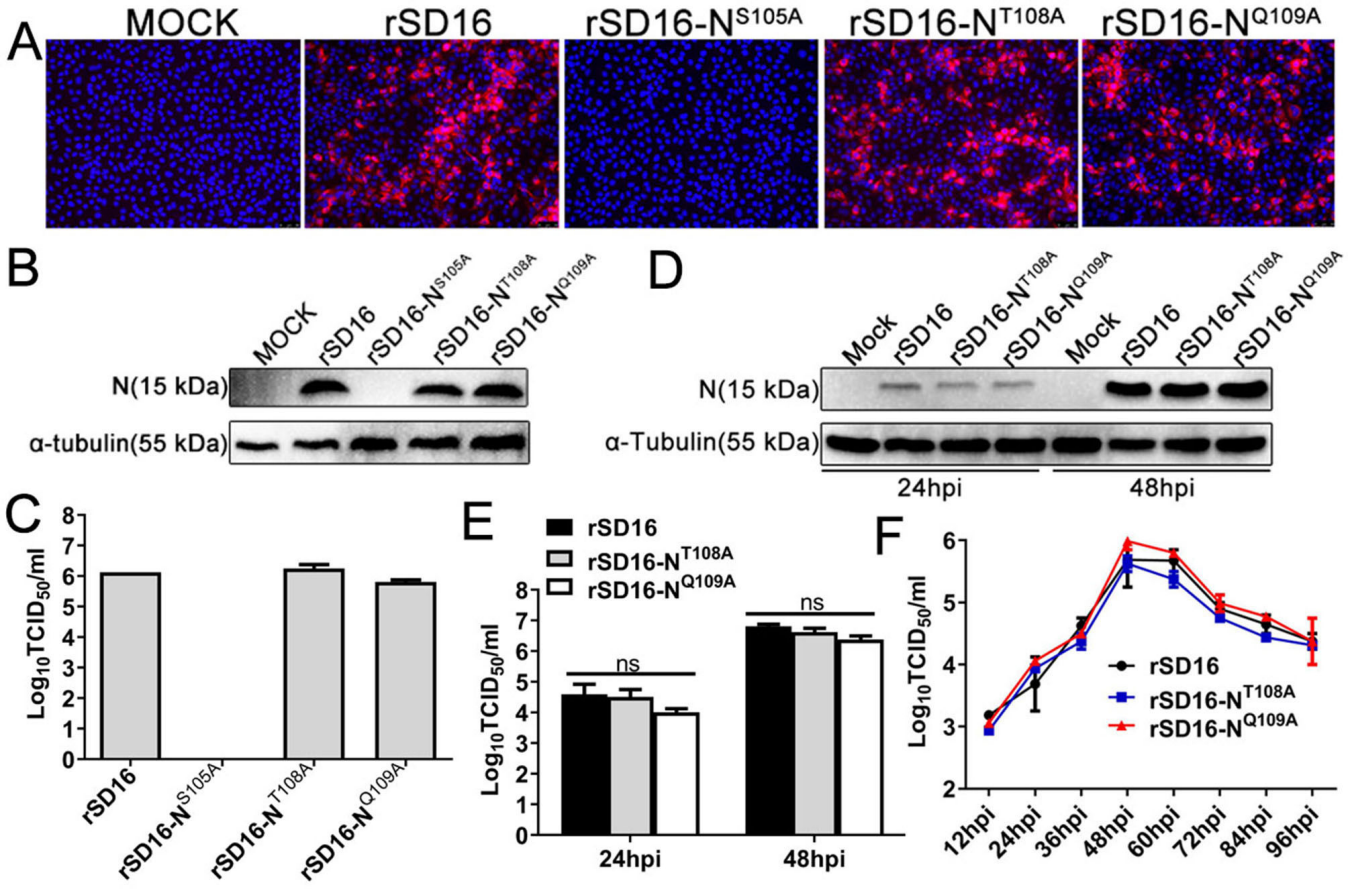
Rescue of different PRRSV infection clones, including the wild-type rSD16 and mutant rSD16-N^S105A^, rSD16-N^T108A^, and rSD16-N^Q109A^. (**A**) Immunofluorescence and (**B**) western blotting analysis of PRRSV N protein in the MARC-145 cells infected with different infection clones. Scale bar, 50 µm. (**C**) TCID_50_ analysis of supernatant from MARC-145 cells transfected with the different PRRSV infection clones. (**D**) Detection of N proteins in MARC-145 cells infected with the mutant rSD16-N^T108A^ and rSD16-N^Q109A^ from the transfected cells cultured for three passages. (**E**) TCID_50_ analysis of the supernatant in MARC-145 cells infected with the mutated rSD16-N^T108A^ and rSD16-N^Q109A^ from the transfected cells cultured for three passages. (**F**) Growth curves of mutant rSD16-N^T108A^ and rSD16-N^Q109A^ in Marc-145 cells. Data are expressed as the mean ± standard deviation of three repeats. Differences between groups were compared using an ANOVA. ns, not significant; PRRSV, porcine reproductive and respiratory syndrome virus; N, nucleocapsid; TCID_50_, 50% tissue culture infective dose.

The above results showed that PRRSV-N-Nb1 inhibited PRRSV replication in MARC-145 and PAM cells, and S105 was the key amino acid for interaction between PRRSV N protein and PRRSV-N-Nb1. Then, the effects of S105 on viral replication were analyzed. The wild-type and mutated PRRSV infection clones, including rSD16, rSD16-N^S105A^, rSD16-N^T108A^, and rSD16-N^Q109A^, were constructed based on the previous descriptions (26). After the four clones were transfected into MARC-145 cells for 72 h, the cell culture supernatants were collected and added to MARC-145 cells for up to three passages. Then, the supernatant from the last passage was used to inoculate MARC-145 cells. At 48 hpi, IFA and western blotting showed that PRRSV N protein was undetected in the MARC-145 cells inoculated with the supernatant from rSD16-N^S105A^ transfection (Fig. 9A and B). The TCID_50_ of progeny virus also showed that no virus was present in the supernatant following rSD16-N^S105A^ transfection (Fig. 9C). These results suggested that the single mutation of S105 to A105 could affect the rescue of PRRSV particles in MARC-145 cells. However, rSD16-N^T108A^ and rSD16-N^Q109A^ could be successfully rescued (Fig. 9A through C), and the two mutations did not affect viral replication (Fig. 9D and E). In addition, growth curves of rSD16, rSD16-N^T108A^, and rSD16-N^Q109A^ were determined in MARC-145 cells. The rSD16-N^T108A^ and rSD16-N^Q109A^ growth rates and maximum titer were similar to those of the parental virus (Fig. 9F).

### Nb1-pFc blocks the self-interaction of PRRSV N protein to inhibit viral replication

**Fig 10:**
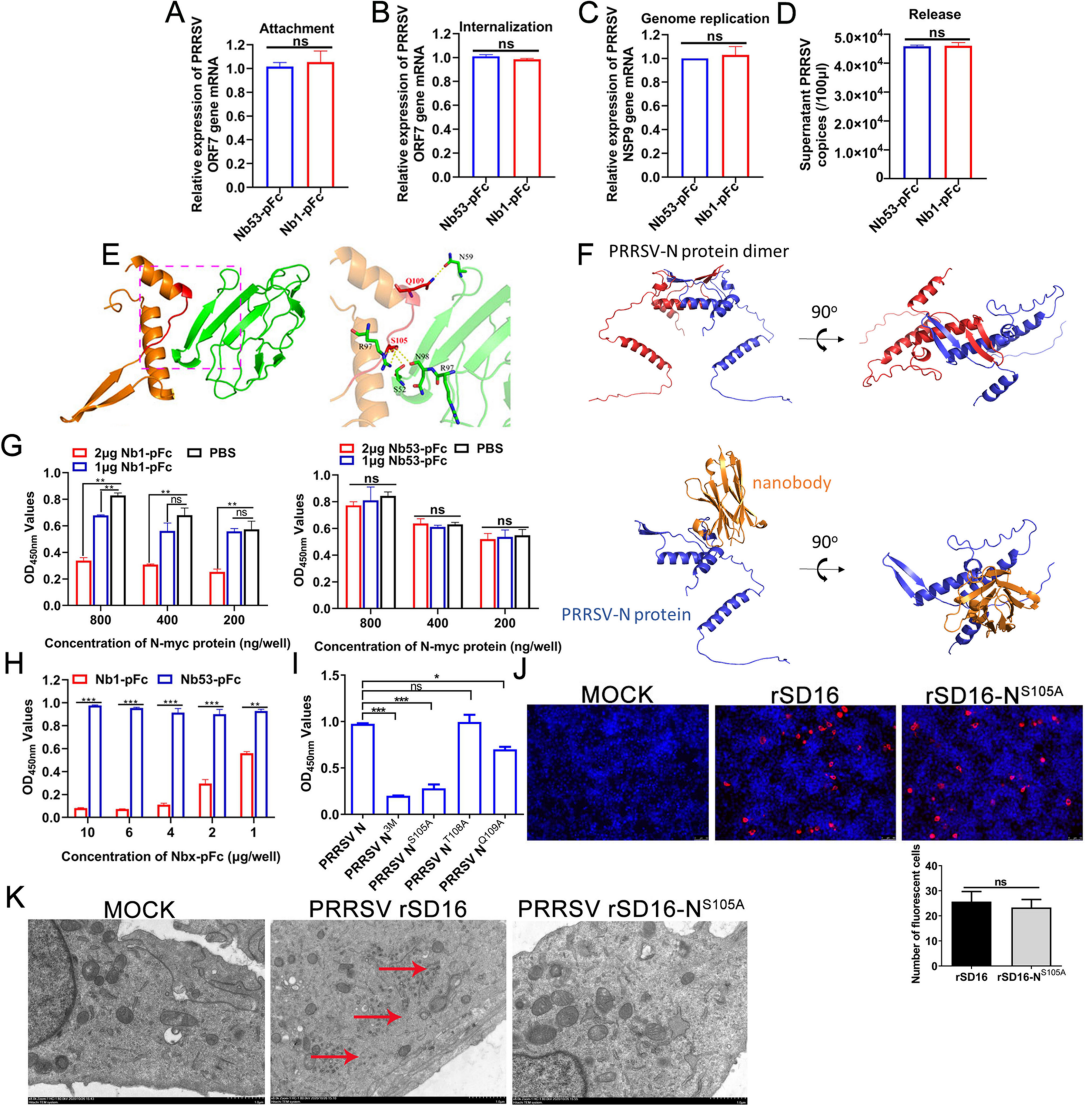
Nb1-pFc blocks the self-interaction of PRRSV-2 N protein. Nb1-pFc did not affect the virus attachment (**A**), entry (**B**), genome replication (**C**), and release (**D**). (**E**) Predicated structure of the docking complex between PRRSV-N-Nb1 and PRRSV N protein. PRRSV N protein is shown in yellow, PRRSV-N-Nb1 is shown in green, and hydrogen bonds are shown by the yellow dotted line. (**F**) Prediction of self-interaction of PRRSV N protein homodimer formation; chains are shown in blue and red. Structure of the predicted docking complex between PRRSV-N-Nb1 and its binding interaction region on PRRSV N protein. PRRSV N protein is shown in blue, and PRRSV-N-Nb1 is shown in yellow. (**G and H**) Blocking ELISA analysis of Nb1-pFc blocking the self-binding of N protein and Nb53-pFc as a control. (**I**) ELISA analysis of binding between N-Myc with mutated PRRSV N proteins (N^3M^, N^S105A^, N^T108A^, and N^Q109A^) with His tag. (**J**) S105A mutation did not affect the N protein expression in HEK293T cells. (**K**) S105A mutation affects viral particle assembly as determined using TEM analysis. Scale bar, 1 µm. Data are presented as the mean ± standard deviation of three repeats. Differences between groups were compared using unpaired Student’s *t*-test. *P < 0.05, **P < 0.01, and ***P < 0.001; ns, not significant; PRRSV, porcine reproductive and respiratory syndrome virus; N, nucleocapsid; TEM, transmission electron microscopy.

To determine Nb1-pFc being involved in the stages of PRRSV replication, the effects of Nb1-pFc on virus attachment, entry, genome replication, and release were first analyzed. The results of RT-qPCR showed that compared with Nb53-pFc, there was no difference in PRRSV ORF7 or NSP9 mRNA in the Nb1-pFc-treated group, suggesting that Nb1-pFc did not affect the virus attachment (Fig. 10A), entry (Fig. 10B), genome replication (Fig. 10C), and release (Fig. 10D). However, based on Fig. 3 showing that Nb1-pFc can significantly inhibit PRRSV replication in PAMs and interact with the N protein, we think that it plays the roles for the late stage (viral assembly) of PRRSV replication.

A previous study showed that the self-interaction of PRRSV N protein was important for encapsulating viral genome replication and viral assembly. So, the effect of Nb1-pFc on affecting self-interaction of PRRSV N protein was analyzed. Firstly, the 3D structures of homology modeling for PRRSV N protein and PRRSV-N-Nb1 were generated using the SWISS-MODE system. Then, the docking interaction model between PRRSV-N-Nb1 and PRRSV-N protein was developed using Discovery Studio Client (version 2.5). The model showed that the S105 forms two hydrogens to bind to aa S52 and N98 sites of PRRSV-N-Nb1. Unexpectedly, a hydrogen bond was also formed between the S105 and R97 sites (Fig. 10E). In addition, the docking models for the interaction of two PRRSV N proteins and between PRRSV N protein and PRRSV-N-Nb1 showed that the PRRSV N protein bound to itself (Fig. 10F), and when PRRSV-N-Nb1 bound to the N protein, it may affect the self-binding of N protein (Fig. 10F). To verify the prediction, a blocking ELISA was performed using PRRSV-N with His tag as the coating antigen, incubated with Nb1-pFc, and then added PRRSV-N with Myc tag. The results showed that the OD_450nm_ values were a reduction of 0.2 to 0.5 in the Nb1-pFc group compared with the Nb53-pFc group, suggesting that Nb1-pFc blocked the binding of PRRSV-N with Myc tag to the coated PRRSV-N with His tag and with a blocking rate of ~60% (Fig. 10G).

Additionally, when the N protein with Myc tag is saturated to 800 ng/well, the Nb1-pFc with 4 µg/well can completely block the self-binding of N proteins (Fig. 10H). To further verify whether the three key binding sites of N protein affect N protein self-binding, PRRSV-N with Myc tag was added to the mutated PRRSV N proteins (N^3M^, N^S105A^, N^T108A^, and N^Q109A^) with His tag as the coating antigen to analyze the combination of four mutated sites with N itself. As shown in Fig. 10I, PRRSV-N^3M^, PRRSV-N^S105A^, and PRRSV-N^Q109A^ proteins can block the self-binding of N proteins.

To further determine the role of S105 in PRRSV replication, the expression of mutated N protein and viral assembly were analyzed. The HEK293T cells were transfected with infection clones rSD16 and rSD16-N^S105A^, and then, the cells were fixed to detect the expression of PRRSV N protein at 48 hpt by IFA. The IFA results showed that PRRSV N protein could be detected in both groups, and there was no significant difference in the expression level (Fig. 10J), indicating that the S105 cannot affect the expression of PRRSV N protein in the process of viral replication. The cell supernatants were collected to infect MARC-145 cells, and the cells were fixed, negatively stained, and observed by TEM. The TEM results showed that the virus particles were observed in MARC-145 cells transfected with the rSD16, but no virus particles were observed in the rSD16-N^S105A^ (Fig. 10K), indicating that the S105 can affect viral assembly.

All the above results indicated that Nb1-pFc cannot affect the viral attachment, entry, genome replication, and release and can block the self-interaction of PRRSV N protein via binding to the motif S105. Meanwhile, the mutated S105 cannot affect the expression of the N protein but affects the viral assembly. In conclusion, we think that Nb1-pFc can enter into the PAMs and bond to the motif S105 of N protein, which blocks the self-binding of N protein following viral assembly and ultimately inhibits virus proliferation.

## DISCUSSION

Due to their ease of production and low cost, nanobodies are considered a promising novel approach for treating diseases. For example, neutralization nanobodies against SARS-CoV-2 have been screened and shown to exhibit efficient antiviral effects (38, 39). Nanobody can also preserve their antigen-binding ability in the reducing environment inside cells (40). So, they can be delivered into cells via some delivery tags to inhibit viral replication. In the present study, two nanobodies against the PRRSV N protein, which were previously identified, were shown to possess favorable antiviral effects *in vitro*. In particular, intracellular expression of PRRSV-N-Nb1 significantly inhibited PRRSV replication in MARC-145 cells. As we know, PRRSV mainly affects pig lung tissue, and nanobody is small in size and easy to transform. Here, we designed the coupling expression of PRRSV-N-Nb1 and pFc, and then, the Nb1-pFc directly targets PAMs. The cytotoxicity of Nb1-pFc was small in PAMs, and the inhibition rate reached more than 90% when the concentration of Nb1-pFc was 40 µM (Fig. 2 and 3).

Additionally, they were shown to possess a broad spectrum of antiviral effects against different PRRSV-2 isolates. Thus, Nb1-pFc may show promise in the treatment of PRRSV-2 infection. PRRSV-2 is common in pig farms in North America and China (41). The Nb1-pFc may serve as the baseline for the design of novel antiviral strategies in combating PRRSV-2 infections in the pig industry of North America and China.

The study also evaluated the inhibition effect of Nb1-pFc on pigs for PRRSV-2 infection. All piglets in the Mock/HP-PRRSV and Nb53-pFc/HP-PRRSV groups died at 12 dpi, with severe pulmonary edema and hemorrhage. However, two pigs survived and recovered in the Nb1-pFc treatment group at 28 dpi (Fig. 4). We believe that the inhibition effect of the Nb1-pFc can be improved by increasing the dose (2 mg–5 mg). In addition, using 1 × 10^6.5^ TCID_50_ of HP-PRRSV JXA1 strain to challenge the pigs, the Mock/HP-PRRSV group showed obvious clinical symptoms at 2 dpi, and all piglets died at 10 dpi. It is considered that a large amount of challenge led to the rapid onset and death of pigs, which may also lead to the partial protection provided by the treatment of Nb1-pFc. In a previous study, all piglets died at 13 dpi using 2 × 10^5^ TCID_50_ of HP-PRRSV RvJXwn strain (42). We speculated that the inhibiting efficiency of Nb1-pFc would be further improved if the virus dose to inoculate the pigs was reduced. In the future, we will continue to explore the protective effect of Nb1-pFc on pigs with different challenge doses. As for long-term and rebound effects, two pigs in the Nb1-pFc treatment group survived at 28 dpi. No pathological changes, such as bleeding and congestion in the lung tissue, were found at autopsy, suggesting that the two pigs had recovered, indicating that there seems to be no long-term and rebound effects of Nb1-pFc. Of course, the long-term and rebound effects will be further evaluated in detail by animal experiments in pigs. In addition, the antiviral effect of the Nb53-pFc group was weak *in vitro* and *in vivo*. According to Fig. 4, compared with the pigs in the MOCK/HP-PRRSV group, the death of pigs is slow, and the pathological changes of the lungs are mild in the Nb53-pFc group. Combined with literature reports, pFc stimulation can upregulate the expression of IL-6, IL-8, IL-10, etc. and inhibit virus replication (29). Collectively, the Nb1-pFc can significantly inhibit the replication of PRRSV-2 *in vitro* and *in vivo*, and then, it can be modified with multivalent nanobodies to see if it can improve its antiviral effect.

Subsequently, it was determined that the conserved region of amino acids 103–109 in PRRSV-2 N protein was the essential domain for binding to PRRSV-N-Nb1. N protein is relatively well conserved compared with other PRRSV proteins, and the amino acid sequence of the same genotype PRRSV N protein was 96%–100% (43). However, they share only ~60% nucleotide similarity between the two genotypes and do not produce cross-protection (44). Previously, five important epitopes in PRRSV-2 N proteins were identified and located at amino acids 30–52, 37–52, 69–112, and 112–123. The amino acid 52–69 region was a common conformational epitope in genotypes 1 and 2 (45–47). In the present study, the epitope (amino acids 103–109) recognized by PRRSV-N-Nb1 was unique to PRRSV-2 and located in the amino acid 69–112 region. In addition, our previous study found that the epitope was an immunodominant one stimulating a strong immune response after PRRSV infection in pigs (24). Therefore, it is hypothesized that the epitope may be used as a target for designing PRRSV-2 marker vaccines by changing the amino acids of the epitope to that of the amino acids in the PRRSV-1 N protein.

The motif S105 of PRRSV-2 N protein was determined to be the key amino acid for interacting with PRRSV-N-Nb1. A previous study documented that the PRRSV N protein forms a double-layer dimer hollowed structure through covalent or non-covalent binding (48). In the present study, homology modeling predicted that the key amino acid S105 also forms a hydrogen bond with itself, the R97 site, in addition to two hydrogen bonds with aa S52 and N98 sites of PRRSV-N-Nb1 (Fig. 10E). The docking modeling of the interaction between PRRSV-N-Nb1 and PRRSV-N proteins showed that PRRSV-N-Nb1 binds to N protein, which blocked self-interaction of PRRSV N protein (Fig. 10F). These results suggested that PRRSV-N-Nb1 binds to PRRSV-2 N protein, which blocks the self-interaction of PRRSV-2 N protein following viral assembly (Fig. 10). The present report is the first to describe nanobody or drug-mediated inhibition of PRRSV proliferation via blockage of N protein self-interaction.

PRRSV N is a multifunctional protein involved in viral replication and pathogenesis (14). The important function of the PRRSV N protein is the formation of nucleocapsid, which participates in covering viral genome and viral assembly (7). A previous study documented that the N protein’s C-terminal region is necessary to maintain conformational integrity and plays an important role in viral assembly (49). Wootton et al. reported that the 11 amino acids at the C-terminus of N protein play a major role in capsid formation and maintenance of conformational structure (50). Our results showed that the S105A mutation affected the self-interaction following viral assembly (Fig. 10). The motif S105 was not located within the region of 11 amino acids described by Wootton et al. In addition, it was also reported that the self-interaction of N protein formed a homodimer, and the dimers are involved in the encapsulating viral RNA genome and assembly of viral nucleocapsid to form infectious viral particles (17, 51).

Interestingly, our results showed that the S105 forms a hydrogen bond with itself, the R97 site, suggesting that the S105 motif of PRRSV-2 N protein may be involved in self-interaction. These results provide novel insights for the molecular basis of PRRSV N self-interaction and a new target for the development of anti-PRRSV drugs. However, whether there are other amino acids that are also involved in self-interaction of N protein remains to be determined.

PRRSV N protein is a phosphorylated structural viral protein (52). In a previous study, it was reported that the motif S105 was a phosphorylation site, and mutation of this site in the PRRSV XH-GD strain resulted in successful rescue, but it affected viral replication (53). This site was mutated in the PRRSV SD16 strain in the present study, and the mutated virus was not successfully rescued. Meanwhile, the PRRSV GD-HD and CH-1a strains were also mutated, and the mutated virus was not successfully rescued (data not shown). Unexpectedly, these differences may be due to the differences among the PRRSV strains. PRRSV-N-Nb1 can bind to the N protein in natural viruses, and no phosphorylation occurred upon expression in bacteria. Thus, the phosphorylation of this site is unlikely to play a role in the interaction between PRRSV-N-Nb1 and N protein. Therefore, we hypothesize that mutation of this site affected the self-interaction of PRRSV-2 N protein and thus affected the viral assembly rather than phosphorylation modification.

**Fig 11:**
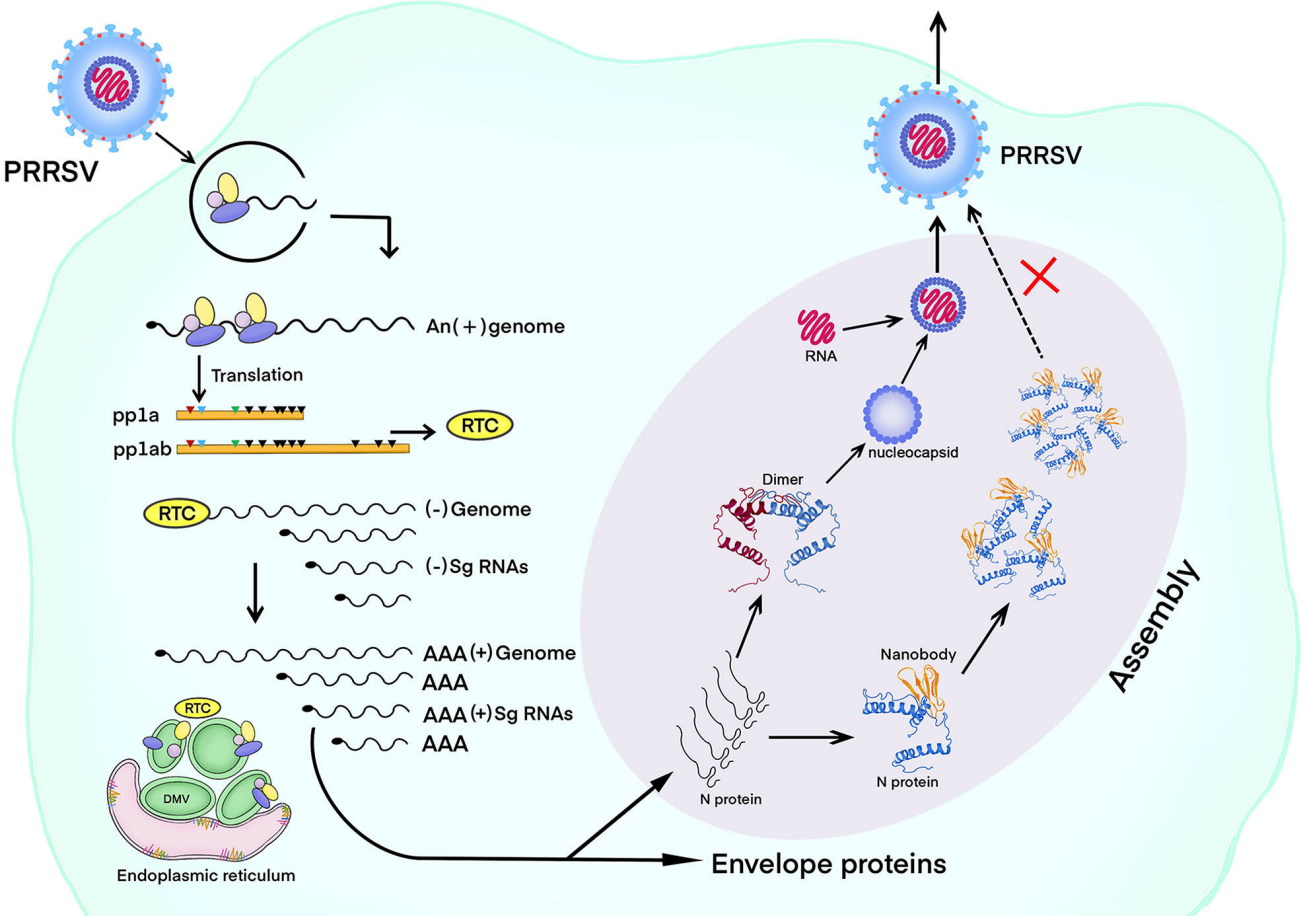
Nanobody targeting PRRSV N blocks itself interaction following viral assembly. A nanobody exhibited a broad-spectrum antiviral effect for PRRSV-2 *in vitro* and *in vivo*. Nanobody targeting S105 motifs of N protein blocks self-interaction of PRRSV N protein, in turn affecting viral assembly. PRRSV, porcine reproductive and respiratory syndrome virus; N, nucleocapsid.

In previous studies, most anti-PRRSV drugs were designed to target non-structural proteins (54, 55). Here, we developed antivirals that target N proteins. The N protein is conserved and is not prone to mutation and drug resistance. The Nb1-pFc has almost no toxicity (endotoxin＜5 EU/mg), and the purity is >95%. Compared with the control group, the side effects of Nb1-pFc were not significantly different in clinical symptoms, rectal temperature, body weight, and food intake after intramuscular injection of 4 mg in 6-week-old pigs (data not shown). Using the HEK293S cells for producing Nb1-Fc, 120 mg protein can be obtained from 1 L culture supernatant, and the production cost is low. In conclusion, PRRSV-N-Nb1 has a good antiviral activity of different PRRSV-2 isolates *in vitro* and *in vivo*. PRRSV-N-Nb1 can block the self-binding of N protein and further prevent the viral assembly via binding to N protein (Fig. 11). The production process of Nb1-pFc protein is simple and low-cost, and it has the value of developing anti-PRRSV drugs.
